# CAM3.0: determining cell type composition and expression from bulk tissues with fully unsupervised deconvolution

**DOI:** 10.1093/bioinformatics/btae107

**Published:** 2024-02-26

**Authors:** Chiung-Ting Wu, Dongping Du, Lulu Chen, Rujia Dai, Chunyu Liu, Guoqiang Yu, Saurabh Bhardwaj, Sarah J Parker, Zhen Zhang, Robert Clarke, David M Herrington, Yue Wang

**Affiliations:** Department of Electrical and Computer Engineering, Virginia Polytechnic Institute and State University, Arlington, VA 22203, United States; Department of Electrical and Computer Engineering, Virginia Polytechnic Institute and State University, Arlington, VA 22203, United States; Department of Electrical and Computer Engineering, Virginia Polytechnic Institute and State University, Arlington, VA 22203, United States; Department of Psychiatry, SUNY Upstate Medical University, Syracuse, NY 13210, United States; Department of Psychiatry, SUNY Upstate Medical University, Syracuse, NY 13210, United States; Department of Automation, Tsinghua University, Beijing 100084, P. R. China; Department of Electrical and Computer Engineering, Virginia Polytechnic Institute and State University, Arlington, VA 22203, United States; Department of Electrical and Instrumentation Engineering, Thapar Institute of Engineering & Technology, Punjab 147004, India; Advanced Clinical Biosystems Research Institute, Cedars Sinai Medical Center, Los Angeles, CA 90048, United States; Department of Pathology, Johns Hopkins University, Baltimore, MD 21231, United States; The Hormel Institute, University of Minnesota, Austin, MN 55912, United States; Department of Internal Medicine, Wake Forest University, Winston-Salem, NC 27157, United States; Department of Electrical and Computer Engineering, Virginia Polytechnic Institute and State University, Arlington, VA 22203, United States

## Abstract

**Motivation:**

Complex tissues are dynamic ecosystems consisting of molecularly distinct yet interacting cell types. Computational deconvolution aims to dissect bulk tissue data into cell type compositions and cell-specific expressions. With few exceptions, most existing deconvolution tools exploit supervised approaches requiring various types of references that may be unreliable or even unavailable for specific tissue microenvironments.

**Results:**

We previously developed a fully unsupervised deconvolution method—Convex Analysis of Mixtures (CAM), that enables estimation of cell type composition and expression from bulk tissues. We now introduce CAM3.0 tool that improves this framework with three new and highly efficient algorithms, namely, radius-fixed clustering to identify reliable markers, linear programming to detect an initial scatter simplex, and a smart floating search for the optimum latent variable model. The comparative experimental results obtained from both realistic simulations and case studies show that the CAM3.0 tool can help biologists more accurately identify known or novel cell markers, determine cell proportions, and estimate cell-specific expressions, complementing the existing tools particularly when study- or datatype-specific references are unreliable or unavailable.

**Availability and implementation:**

The open-source R Scripts of CAM3.0 is freely available at https://github.com/ChiungTingWu/CAM3/(https://github.com/Bioconductor/Contributions/issues/3205). A user’s guide and a vignette are provided.

## 1 Introduction

Complex tissues are dynamic ecosystems comprised molecularly distinct cell types that collectively orchestrate tissue phenotypes Hart *et al.* (2015). To understand many biological processes affecting diseases, it is critical to identify specific driver cell types and their composition in native microenvironments Herrington *et al.* (2018). However, these latent features are typically not revealed when a heterogeneous sample is studied in bulk.

Computational deconvolution provides a cost-effective technique to dissect bulk data into cell type composition and expression, and has the potential of extracting novel yet reproducible biological information from vast amounts of existing multi-omics bulk data, resulting in gains in statistical power and research opportunities for a wide range of previous experimental designs (Rahmani *et al.* 2019). With few exceptions (Supplementary Table S11), most existing deconvolution tools exploit supervised approaches requiring various types of references (cell types, mixing proportions, marker genes, or signature expressions) that may be unreliable or even unavailable for specific tissue microenvironments (Avila Cobos *et al.* 2018, Sutton *et al.* 2022). Thus, supervised deconvolution is poorly suited to determining cell type composition and expression that are condition-specific or previously unknown.

To complement supervised approaches, a few unsupervised deconvolution methods have been proposed with the potential advantage of capturing a more complete representation of cell types and states under different biological conditions (Chan *et al.* 2008, Hart *et al.* 2015, Houseman *et al.* 2016, Wang *et al.* 2016, Li and Wu 2019, Zhang *et al.* 2021). More importantly, unsupervised deconvolution tools are conceptually applicable to a much broader array of datatypes such as metabolomic or medical imaging data where references for biological processes are commonly lacking (Chen *et al.* 2011). Furthermore, unsupervised deconvolution can extract biologically interpretable latent features at not only cellular but also tissue or biological task levels (Hart *et al.* 2015, Fan *et al.* 2020).

**Figure 1.**
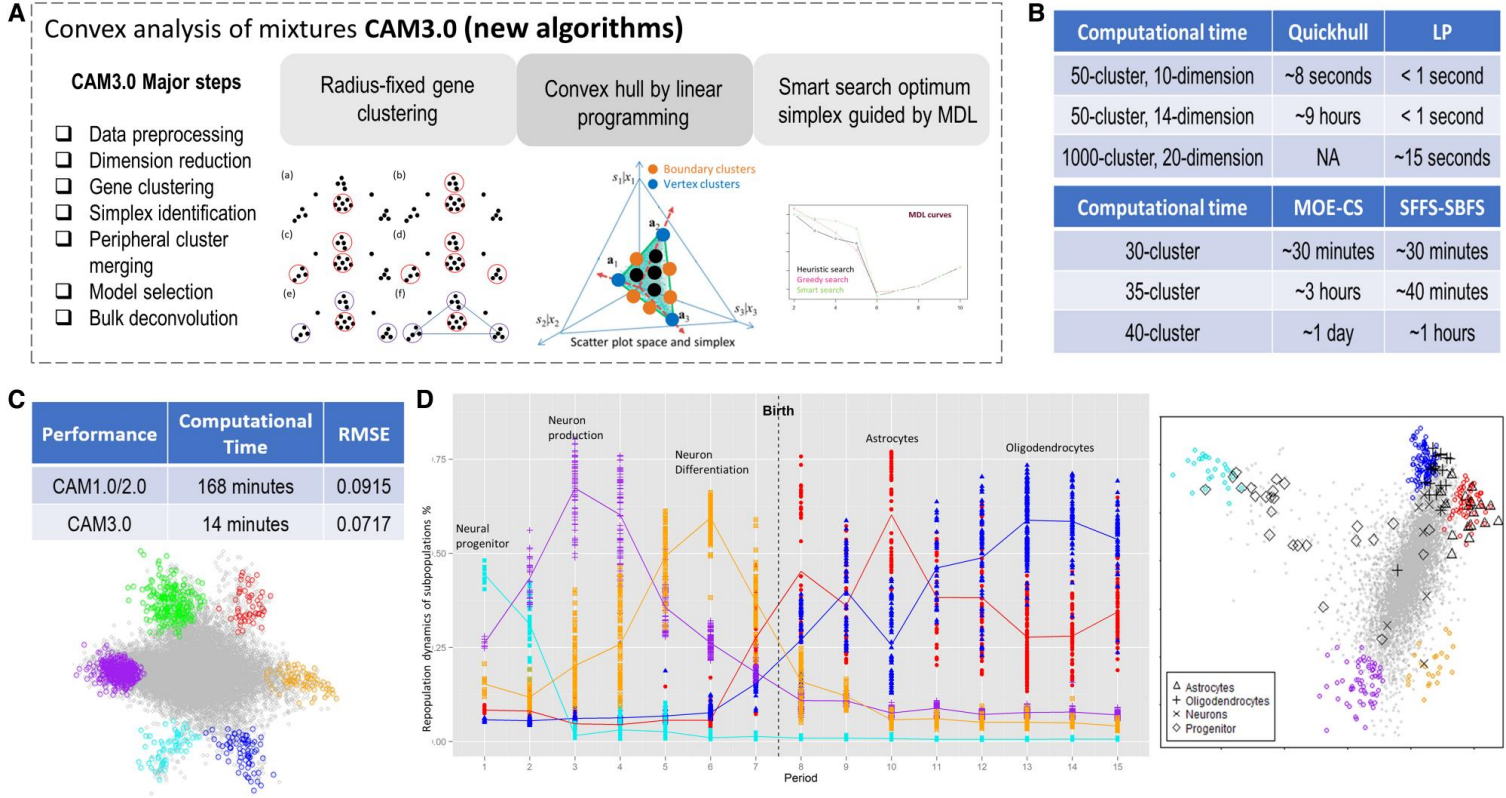
Overview of CAM3.0 tool. (**A**) Schematic representation of CAM3.0 workflow (overall seven-step pipeline and three new algorithms). (**B**) Computational efficiency of the new algorithms for identifying the initial scatter simplex and optimum latent variable model. (**C**) Improved accuracy and efficiency of CAM3.0 tested on the benchmark datasets (GSE64385). (**D**) Biomedical case study on reconstructing the cellular repopulation dynamics during the human brain development and lifespan, directly from the time-courses of the bulk expression data (GSE25219).

Here we introduce CAM3.0, an open-source software tool to determine cell type composition and expression from bulk tissues under diverse biological conditions. To accomplish this, we improved the Convex Analysis of Mixtures—CAM, a fully unsupervised deconvolution framework that we previously developed (Chan *et al.* 2008, Wang *et al.* 2016), with three new and highly efficient core algorithms (radius-fixed clustering, linear programming, smart floating search) (Fig. 1A), that can assist biologists to more accurately identify reliable cell markers, determine cell proportions, and estimate cell-specific expressions. We demonstrate the effectiveness and utility of these new functions in CAM3.0 using both realistic simulations and real biomedical case studies, showing improved accuracy of data deconvolution compared with relevant peer methods.

## 2 Materials and methods

### 2.1 Review of the CAM framework and previous applications

Convex analysis of mixtures considers a convex-set latent variable model X=AS, where X is the observed bulk expression matrix, A is the sample-wise cell type proportion matrix, and S is the cell type specific expression matrix. Fundamental to the success of CAM are the mathematical theorems showing that the scatter simplex of bulk expressions is a rotated and compressed version of the scatter simplex of cell type specific expressions, where cell type markers are located at each of the vertices precisely corresponding to the column vectors of the cell type proportion matrix A (Fig. 1A) (Chan *et al.* 2008, Wang *et al.* 2016). As a fully unsupervised deconvolution method, CAM resolves the cell type proportion matrix A by geometrically identifying the vertices of the bulk expression scatter simplex, and subsequently estimates the cell type specific expression matrix S. The minimum description length (MDL) determines the number *K* of constituent cell types. We and others have previously demonstrated the performance and utility of CAM method on gene expression (Hart *et al.* 2015, Wang *et al.* 2016, Dong *et al.* 2020), proteomics (Herrington *et al.* 2018, Parker *et al.* 2020), imaging (Chen *et al.* 2011, Fan *et al.* 2020), and methylation data (Chen *et al.* 2020).

Below we briefly describe the principles of three newly developed core algorithms in CAM3.0 (Supplementary information), namely, radius-fixed clustering (RFC), linear programming (LP), and smart floating search (SFS). For readers interested in the mathematical formulation, algorithmic workflow, and comparative evaluations of the CAM approach versus peer methods, we highly recommend the original reports (Wang *et al.* 2016, Chen *et al.* 2020) and comprehensive reviews (Avila Cobos *et al.* 2018, Sutton *et al.* 2022).

### 2.2 Radius-fixed clustering to identify reliable markers

Cell markers play a critical role in resolving the cell type proportion matrix A for unsupervised deconvolution (Avila Cobos *et al.* 2018). To detect reliable cell markers, in our previous CAM workflow, we adopted the popular k-mean clustering (KMC) method that partitions the scatter simplex into Voronoi clusters. However, KMC often produces non-unique clusters of uneven sizes and densities including the most sought-after vertex clusters. KMC is also sensitive to outliers, random initialization, and manually assigned cluster numbers.

In CAM3.0, we propose a deterministic RFC method that can effectively identify high-quality marker clusters with sufficient tightness and density (reduced uncertainty), yet achieving a unique global optimum. With properly chosen thresholds on cluster radius and minimum membership number, RFC starts every gene vector as a cluster center, and calculates cosine scores from other genes to aggregate neighbor genes within the specified radius. Iteratively, RFC identifies the top cluster with the largest number of member genes, removes the associated genes, and performs the next round of clustering until no qualified cluster can be found (Fig. 1A). Also, RFC can automatically determine the total number of high-quality clusters and detect no-membership genes as outliers.

### 2.3 Linear programming to detect an initial scatter simplex

To detect the initial scatter simplex that most tightly encloses all gene clusters, in our previous CAM workflow, we adopted one of the benchmark methods—Quickhull (Chen *et al.* 2020), but at the cost of a significantly high computational complexity particularly when sample size is large (scatter dimension). Inspired by the very definition of a convex-set, where any interior point is a non-negative combination of all points while exterior clusters (vertices) must be a trivial non-negative combination, in CAM3.0 we formulate the detection of all vertex clusters as a linear programming (LP) problem,

(1) min⁡sj; s.t. ∑i=1Nsixi=xj, ∑i=1Nsi=1, si≥0, 

where an $x_{j}$ with a non-zero weight $s_{j}$ would be a vertex cluster and *N* is the total number of clusters. The LP strategy has a much shorter computational time than that of Quickhull (weak polynomial), and can test all clusters simultaneously (parallel computing). Thus, this new detection scheme makes CAM3.0 broadly applicable to much larger datasets.

### 2.4 Smart floating search for the optimum latent variable model

To determine the optimum number *K* of constituent cell types, an exhaustive search for the optimum simplex with lowest MDL presents a high computational burden particularly when *K* is large (Chen *et al.* 2020). In CAM3.0, we propose an SFS with three effective strategies. First, we offer an option to merge adjacent clusters before the floating search that will lower not only the search burden, but also instability due to ill-conditioned simplex matrices. Second, we combine Sequential Forward Floating Search (SFFS) and Sequential Backward Floating Search (SBFS) schemes to survey the MDL optimality of simplex for every *K*, significantly reducing the search complexity. Third, we approximate the reconstruction error by the “unexplainable portion” of the competing floating simplex and remove the vertex candidate with the lowest residue-error contribution, further cutting down the computational time.

## 3 Results

### 3.1 Validation on realistic simulation data

We assessed the performance of these new functions using realistic simulations. We applied RFC to a real gene expression dataset (3 cell types, 30 000 genes). The experimental results show that RFC yields more unique solutions with higher density, reduced outlier contamination, and proper cluster numbers compared with KMC (Supplementary Information). We tested LP based detection of an initial scatter simplex, in comparison with Quickhull, on the simulated data with varying numbers of clusters and dimensions. LP required significantly shorter computational time, particularly for larger sample sizes, compared with Quickhull (Fig. 1B). Finally, we compared the smart floating search against a combinatorial search for the optimal simplex solution. The required computation times are given in Fig. 1B, showing that the smart floating search is much faster than a combinatorial search in finding the optimal simplex, particularly for larger cluster numbers.

### 3.2 Comparative evaluation on benchmark and simulation data

We evaluated the efficiency and accuracy of CAM3.0, in comparison with the earlier CAM tools (Wang *et al.* 2016, Chen *et al.* 2020), on a benchmark gene expression dataset (GSE64385: 6 cell types, 54 675 genes). The 12 samples represent the biological mixtures of five immune cell subtypes and one cancer cell line in known proportions. CAM3.0 used only 14 min while earlier CAM tools used 168 min to complete the same task. The optimal scatter simplex and associated marker clusters, blindly identified by CAM3.0, are given in Fig. 1A and C, showing six distinctive vertices. Also, CAM3.0 achieved a higher accuracy in estimating the proportion matrix A than that of earlier CAM tools, measured by the root-mean-square-error (RMSE) between the estimate and ground truth (Fig. 1C). We also compared the performance of CAM3.0 and the four most relevant peer methods using the ground truth embedded realistic simulations generated from three benchmark gene expression (GSE19380 and GSE73721) and DNA methylation (GSE110554) datasets. The experimental results show that CAM3.0 consistently outperforms the three unsupervised methods with higher accuracy of estimating cell type proportion matrix (Supplementary Tables S12–13, S15–17) (Houseman *et al.* 2016, Li and Wu 2019, Zhang *et al.* 2021); and also outperforms the one supervised method in estimating cell type specific gene expression (Supplementary Table S14) when the supervising information is less accurate or incomplete (Rahmani *et al.* 2019).

### 3.3 Interpretable biomedical case study

We applied the CAM3.0 tool to the benchmark human brain lifespan dataset, Human Brain Transcriptome (HBT) (GSE25219, *n* = 923). The time-courses of bulk gene expressions were sampled over human brain developmental periods and lifespans (Fig. 1D). CAM3.0 identified the optimum latent variable model with five vertices, where the *de novo* vertex-resident markers match well with the *a priori* markers associated with the cell types in the human brain. CAM3.0 then reconstructed the repopulation dynamics (as a function of time) of five molecularly distinct cell types or states and replicated the results in both region-specific HBT and independent Braincloud (GSE30272, n = 269) datasets (Supplementary Information). These results are biologically plausible and match well with the previously-validated repopulation dynamics data about human brain development and lifespan (Supplementary Information).

## 4 Discussion

In this application note, we present CAM3.0 as a fully unsupervised and broadly applicable deconvolution tool intended to complement existing reference-free or reference-based methods. For example, the outcomes of CAM3.0 can readily provide complementary references for supervised or semi-supervised deconvolution methods when study- or datatype-specific references are unreliable or unavailable (Rahmani *et al.* 2019, Dong *et al.* 2020). Key features that distinguish it from previous work include three novel and highly efficient algorithms that make it effective and robust to examine larger and more complex datasets yet achieve improved accuracy (Houseman *et al.* 2016, Li and Wu 2019, Zhang *et al.* 2021). CAM3.0 identifies tightly-clustered cell type markers in scatter space to define biologically interpretable latent features without requiring the existence of pure samples in the sample-space (Hart *et al.* 2015). The selection of the two hyperparameters (cluster radius and min membership number) remains a topic for future research.

## Supplementary Material

btae107_Supplementary_Data
