# Supplementary material for: CAM3.0: determining cell type composition and expression from bulk tissues with fully unsupervised deconvolution
Source: Bioinformatics. 2024 Feb 26;40(3):btae107. doi: 10.1093/bioinformatics/btae107 (PMC10924278; doi:10.1093/bioinformatics/btae107)
Supplement: btae107_Supplementary_Data [file btae107_supplementary_data.docx]

Supplementary Information

**CAM3.0: Determining cell type composition and expression from bulk tissues with fully unsupervised deconvolution**

Chiung-Ting Wu, Dongping Du, Lulu Chen, Rujia Dai, Chunyu Liu, Guoqiang Yu, Saurabh Bhardwaj, Sarah J. Parker, Zhen Zhang, Robert Clarke, David M. Herrington, and Yue Wang

**Contents**

[Methods 2](#_Toc155960954)

[Results (with supplementary figures and tables) 15](#_Toc155960955)

[Experimental comparison with peer methods 28](#_Toc155960956)

[Discussion 37](#_Toc155960957)

[R Scripts 42](#_Toc155960958)

[References 43](#_Toc155960959)

## Methods

**Background and Motivation**

Complex tissues are characterized by productive interactions between different cell types and cell-specific sub-phenotypes to achieve specialized biologic functions (Lake, et al., 2016). Documenting the identity and dynamic composition of these cell types and their expression is important to understand normal biological processes and disease pathogenesis. For example, shifts in the relative composition of neuron or glia cells is central to the developmental processes of the human brain (Colantuoni, et al., 2011; Kang, et al., 2011). Likewise, changes in cell-specific molecular expressions is of direct etiological relevance for the pathogenesis of atherosclerosis (Herrington, et al., 2018). Biologists have amassed a large body of information about molecular remodeling of complex tissues under different conditions (Dadgar, et al., 2014). However, most of our knowledge relies on mixed readouts of bulk tissues. An experimental solution to mitigate the challenge of cellular heterogeneity contributing to these readouts is to sort cell types or isolate individual cells before molecular profiling. While promising, physical separation is clearly not the most reliable and cost-effective method and is inapplicable to previously-assayed samples (Avila Cobos, et al., 2018). Moreover, the effects of some important cell-cell and cell-matrix interactions present in complex tissues may be lost in samples of experimentally separated cells. Computational deconvolution of readouts from bulk tissues may overcome some of these limitations and provide a complementary tool to use in the study complex tissues.

Computational deconvolution of bulk data includes three sub-tasks: (1) detect the presence of, and the uniquely identifying molecular makers for each cell type present in the tissue samples, (2) determine cell type proportions in individual samples, and (3) estimate cell specific expression (Avila Cobos, et al., 2020; Avila Cobos, et al., 2018; Chen, et al., 2020; Jaakkola and Elo, 2021; Wang, et al., 2016). In the past, supervised methods for deconvolution have been developed that make use of externally derived cell specific markers or references. However, supervised methods are poorly suited to find molecularly distinct cell types that are subtle, condition-specific (their distinctive signatures change when the cells are present in different microenvironments), or previously unknown (Houseman, et al., 2016; Kuhn, et al., 2011; Newman, et al., 2015). The reported experimental evaluations using single-cell RNA-sequencing data show that supervised deconvolution using general references for specific studies often produces inaccurate estimates, and substantially worse results when the references are less accurate with respect to a specific study or it fails to include all constituent cell types in the reference (Chen, et al., 2022; Wang, et al., 2021) (Table S14, Figure S13, Figure S14).

More recently, fully unsupervised deconvolution methods have been developed that provide a more comprehensive and unbiased approach to the characterization of the molecular landscape of complex tissues. Supported by advanced machine learning algorithms and proven mathematical theorems, unsupervised deconvolution methods can decompose the mixed molecular signals into multi-level latent variables (tissue types, cell types, cell states, biological processes, biological tasks) that are biological interpretable and functionally enriched (e.g., molecular markers and signatures) (Gaujoux and Seoighe, 2012; Hart, et al., 2015; Herrington, et al., 2018; Houseman, et al., 2016; Krug, et al., 2020; Moffitt, et al., 2015; Wang, et al., 2016).

**Review of previous CAM framework development**

Here we review the underlying conceptual framework for CAM and summarize our previous work to develop CAM as a tool for unsupervised deconvolution. The CAM deconvolution workflow is built on the strong parallelism between linear latent variable models and the theory of convex sets (Chan, et al., 2008; Chen, et al., 2011; Wang, et al., 2016). Bulk samples to be analyzed by CAM contain an unknown number and varying proportions of molecularly distinctive cell types (including cell states or biological tasks). Molecular expression in a specific cell type is modeled as being linearly proportional to the abundance of that cell type. Applying the newly-proven mathematical theorems (Wang, et al., 2016), we showed that the simplex of mixed expression patterns in bulk tissues, in the scatter space of the mixtures (for example, gene expression scatter space), is a rotated and compressed version of the simplex of subtype expressions. According to the theory of convex sets (Chan, et al., 2008), every molecular feature within the scatter simplex can be uniquely determined by the linear combination of the vertices. Thus, the number of the vertices corresponds to the number of molecularly distinctive cell types present in the bulk samples and the molecular features residing at the vertices are the molecular markers defining such cell types or biological tasks (Wang, et al., 2016).

The CAM deconvolution framework works by detecting the vertices of the scatter simplex geometrically, *i.e.*, determining the multifaceted simplex that most tightly encloses the bulk expressions. Subsequently, we identify the molecular markers residing at the vertices, and estimate the proportions and specific expression profiles of constituent cell types (Wang, et al., 2016). The number of cell types present is determined by the newly-derived minimum description length (MDL) criterion (Chen, et al., 2011; Lin, et al., 2018; Wang, et al., 2016). Ideally, a molecularly distinctive cell type would contain molecular signatures (molecular markers) that are exclusively expressed in the cognate cell or tissue type of interest while in no others. Importantly, our deconvolution pipeline requires no a priori information on the number, signatures, or compositions of the cell types present in bulk samples, and does not require the presence (existence) of pure cell type samples, see the illustrative ternary plot (Hart, et al., 2015; Schwartz and Shackney, 2010). This advantage is significant in that CAM can achieve all of its goals using only a small number of bulk samples, and provides a powerful means to distinguish among phenotypically similar cell types.


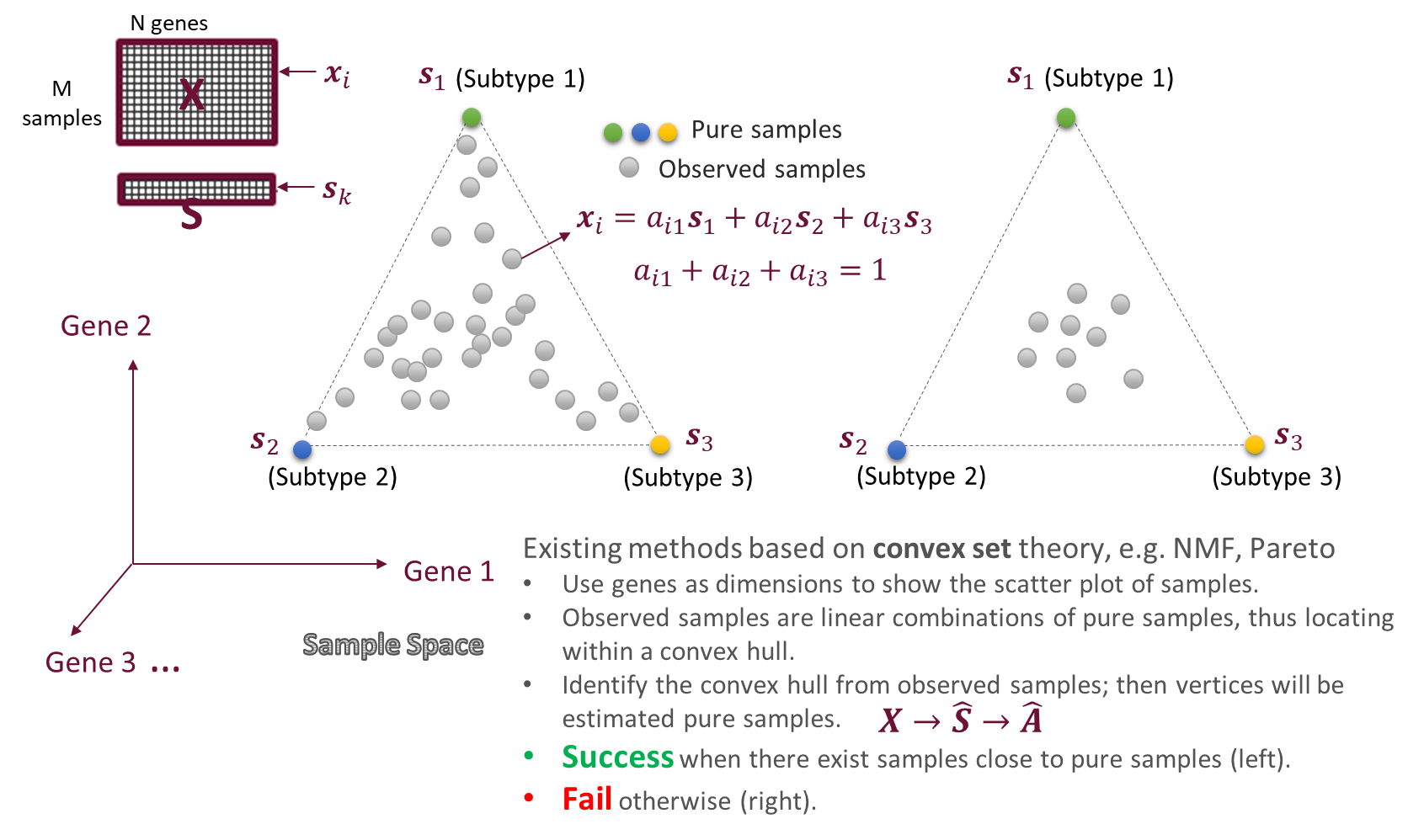


While there may be many ways to subdivide a complex tissue ecosystem, there is clearly a need to dissect the complexity of many multiscale molecular landscapes. We have shown the performance and biomedical utility of CAM based deconvolution using gene expression (Wang, et al., 2016), methylation (Chen, et al., 2020), proteomics (Herrington, et al., 2018; Parker, et al., 2020), and imaging data (Chen, et al., 2011; Chen, et al., 2011; Fan, et al., 2020). These applications have led to novel findings and hypotheses. A molecular latent variable model of complex tissues, particularly in the presence of disease lesions, is not yet available, but we can provide a roadmap of how a machine learning approach might uncover, in mathematical forms, the molecular events controlling tissue remodeling in many biomedical contexts. The value of this deep deconvolution is illustrated by the fully unsupervised learning outcomes obtained from molecular expression data of complex tissues, and will be measured ultimately by the emerged new insights or hypotheses.

**Review of mathematical modeling in previous CAM workflow**

Here we formulate the deconvolution task as a blind source separation (BSS) problem (Chan, et al., 2008). Supposing the number of samples (bulk samples) is *M*, number of genes (features) is *N*, and number of cell types is *K*, the matrix factorization problem can be stated as (**Fig. S1**)

$\mathbf{X=AS}, (1)$

where $\mathbf{X}$ is a $M\times N$ gene expression matrix, where each row corresponding to a bulk sample, and each column corresponding to a gene (feature). $\mathbf{A}$ is a $M\times K$ mixing matrix, where each row corresponding to a bulk sample, and each column corresponding to a molecular cell type. That is, each row in $\mathbf{A}$ represents the proportion of each cell type in a sample. $\mathbf{S}$ is a $K\times N$ source matrix, where each row corresponding to a cell type, and each column corresponding to a gene. Thus, each row in $\mathbf{S}$ represents the expression in a cell type. Our target is to estimate $\mathbf{A}$ and $\mathbf{S}$. The only information available is the data matrix $\mathbf{X}$, so this is a blind source separation problem.


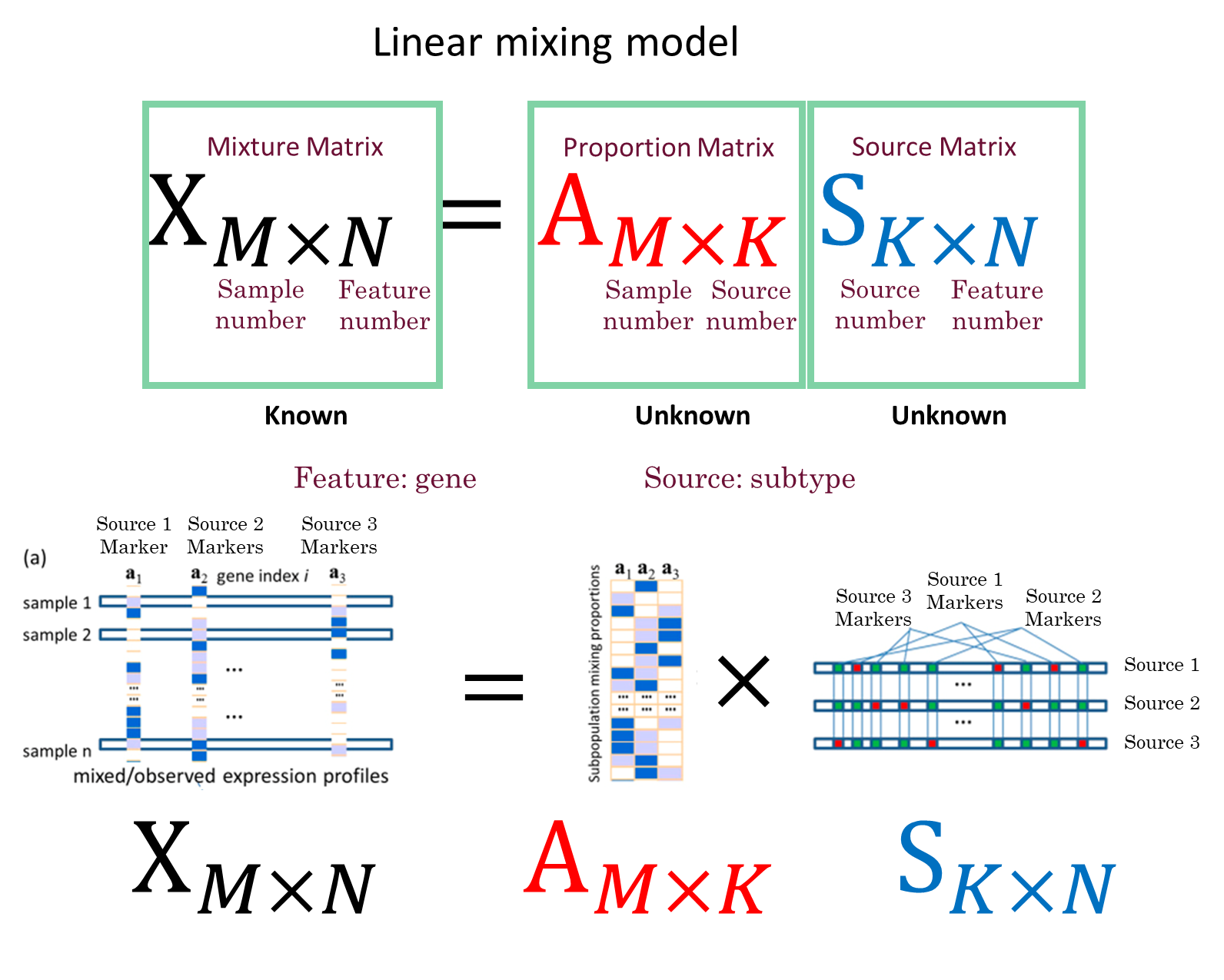


**Figure S1**. Illustration of linear mixing model in relation to the underlying latent variable model.

We assume that for each dimension (each cell type), there is at least one gene as the Cartesian vector (marker gene), though the length is not one (**Fig. S1**). Thus, if we normalize all the genes by their expression sum (the column sums are all 1 after normalization), all the genes are the convex combinations of the marker genes from each cell type. That is, the normalization projects the genes onto a hyper plane, where the genes form a (*K*-1)-simplex, and the marker genes are on the vertices of the simplex. Thus, if we multiply $\mathbf{A}$ and $\mathbf{S}$, which is similar to rotation and projection of the (*K*-1)-simplex onto a higher dimensional space (**Fig. S2**), and the vertices of the simplex are exactly the column vectors of $\mathbf{A}$. That is, after projection, the column vectors (genes) in $\mathbf{X}$ can be viewed as a convex combination of the column vectors in $\mathbf{A}$, and the vertices of the simplex are exactly the vectors in $\mathbf{A}$. Thus, after projecting the column vectors (genes) of $\mathbf{X}$, we just need an algorithm, such as Quickhull (Barber, et al., 1996), to find the convex hull, and points on the convex hull are the candidates of the vertices (**Fig. S3**). See below illustration of the relation between S and X (K = 3 for example). The upper left corner is an example of sum-to-1 normalization in 2D space, which project the genes onto 2 1-simplex (a line). The red dots are the marker genes, and the purple dots are the normal genes. The right part is the simplex in 3D space (a triangle). After multiplying with A, the simplex is rotated and projected into a higher dimension space (four in this figure), and the vertices (markers) become the column vector of A, as shown in the figure.


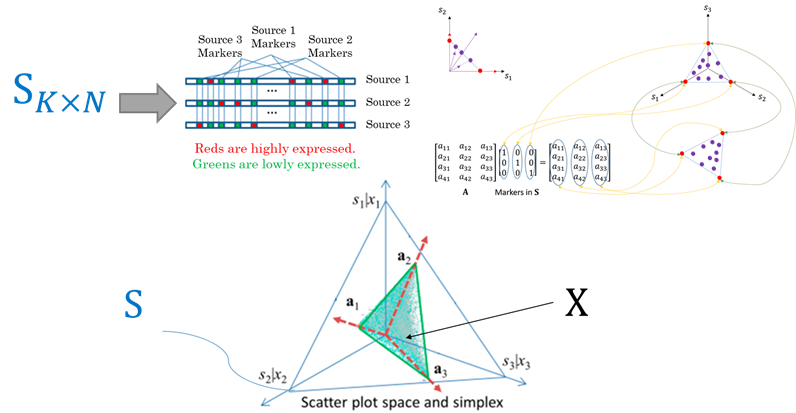


**Figure S2**. Geometric illustration of linear mixing model in scatter space.

CAM applies Minimum Description Length (MDL) (Wax and Kailath, 1985) to find the optimal *K*. MDL is a widely-accepted model selection method, which is rooted in the information theory. The concept of MDL is to find the balance between low reconstruction error (estimation bias) and low model complexity (estimation variance). The MDL in CAM is formulated as:

$$\begin{aligned} MDL\left( K \right)=\frac{1}{2}\log\left( \sum_{j=1}^{N} \left\| \boldsymbol{x}\left( j \right)-\mathbf{A}^{'}\mathbf{s}^{'}\left( j \right) \right\|_{2}^{2} \right)+\frac{\left( K-1 \right)M}{2}\log\left( N_{MG} \right)+\frac{KN}{2}\log\left( M \right),\#\left( 2 \right) \end{aligned}$$

where the first term corresponds to the model fitting error (reconstruction error), and the second and the third terms corresponding to the model complexity, all measured in terms of the average bits required to encode the information. That is, MDL is trying to find a model (*K*) where the reconstruction error is low (first term), and the number of sources (*K*) is also low (second and third terms). We can also view the second and the third terms as penalty terms, which are used to ease the overfitting problem. With MDL, unlike other BSS methods (ICA/nICA, NMF, Pareto) (Gaujoux and Seoighe, 2012; Gaujoux and Seoighe, 2013; Hart, et al., 2015; Houseman, et al., 2016), CAM is a fully unsupervised deconvolution method, since the value of *K* could be estimated by CAM automatically, which is not provided by other most relevant peer methods (**Figs. S3-4**).


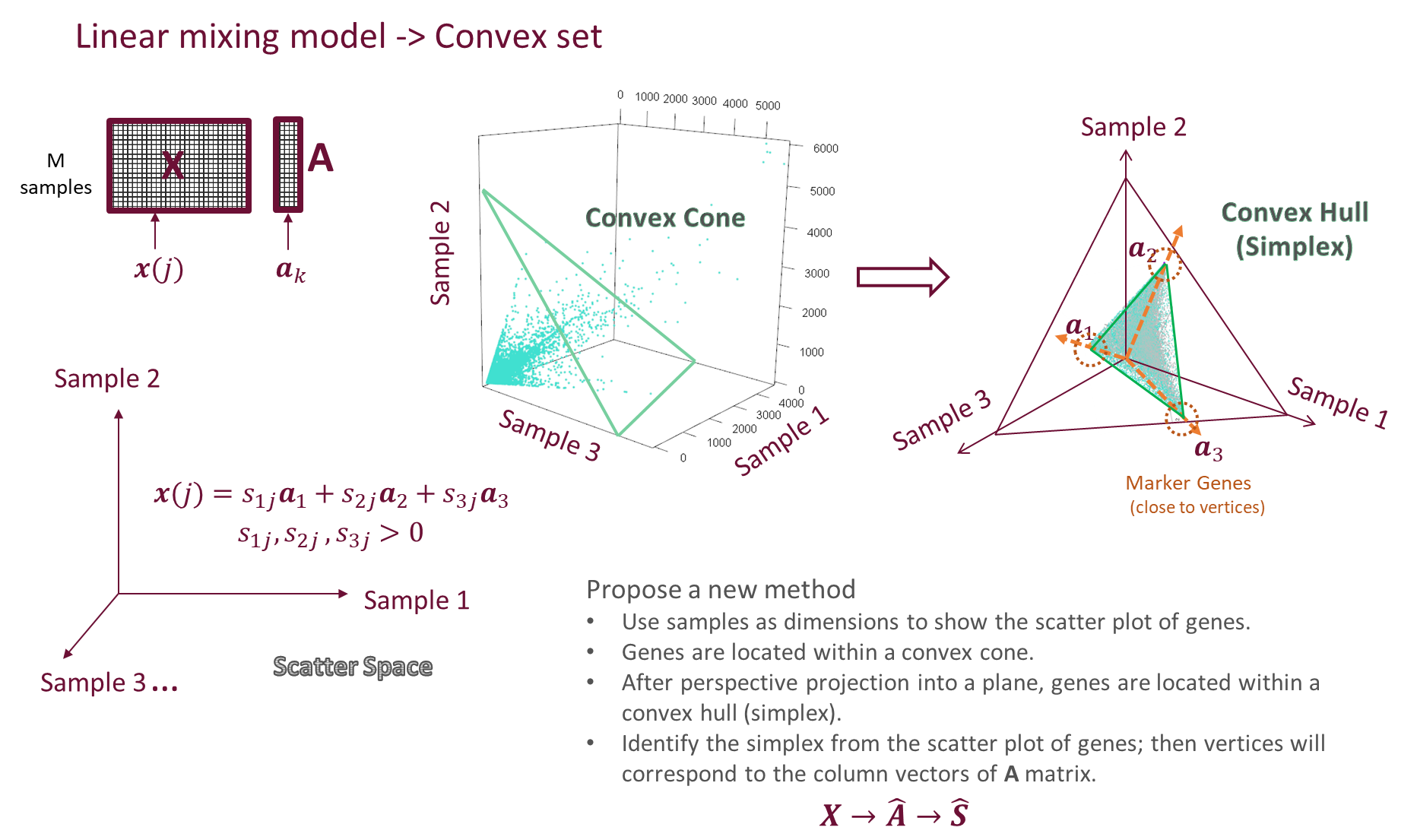


**Figure S3**. Illustration of the CAM framework.


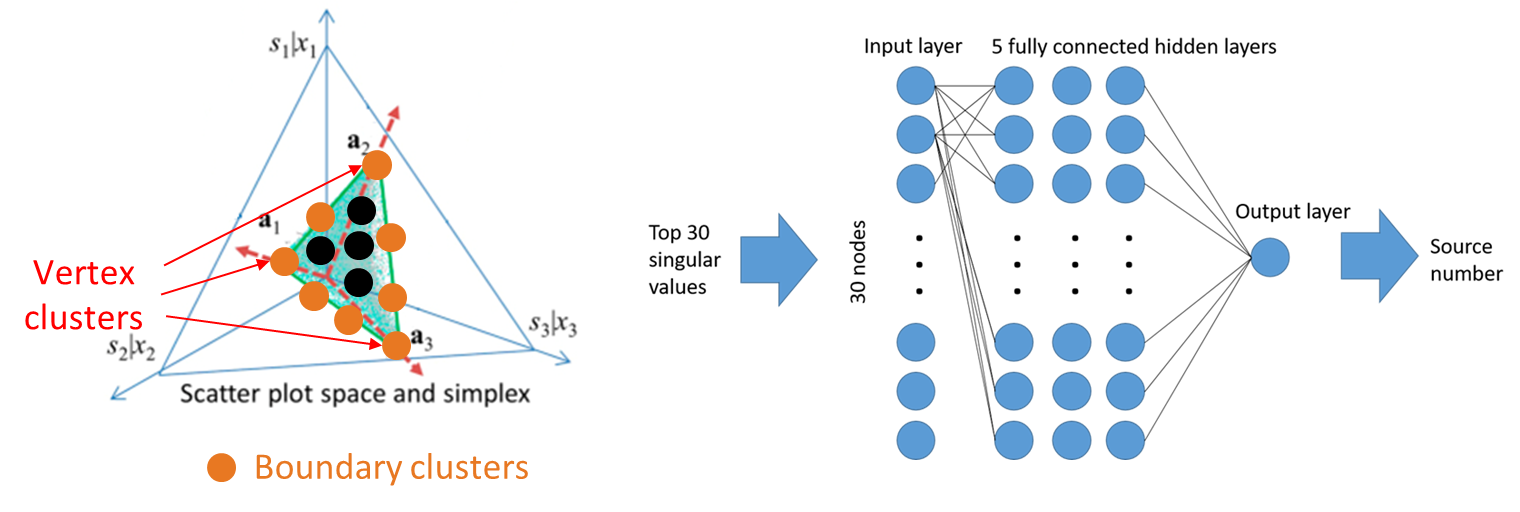


**Figure S4**. Deep-learning guided model selection to identify the optimum scatter simplex.

**Motivation and detailed description of the novel algorithms developed in CAM3.0**

Below we describe in details the principles and pipelines of three newly developed core algorithms in CAM3.0, i.e., radius-fixed clustering (RFC), linear programming (LP), and smart floating search (SFS). The major problems associated with previous CAM workflow include: (1) unsatisfactory quality assurance on gene clustering specifically related to marker gene clusters, (2) high computational complexity in finding scatter convex hull, and (3) high computational complexity in identifying the optimal simplex of latent variable model. To efficiently address these problems, in the CAM3.0 development, we propose a deterministic radius-fixed clustering (RFC) method that can effectively assure the quality of marker clusters with sufficient tightness and density (reduced uncertainty) with two algorithmic parameters (*radius.thres, MG.num.thres,* jointly ensure the quality of all clusters and also help terminate the clustering iterations), yet achieving a unique global optimum; we then formulate the detection of all peripheral clusters as a linear programming (LP) problem that has a much shorter computational time than that of Quickhull (weak polynomial), and can test all clusters simultaneously (parallel computing); we also propose a smart floating search (SFS) with three effective strategies particularly when the number of constituent cell types is large (*sim.thres, cluster.num*).

**Radius-fixed clustering** (for feature down-sampling and quality marker clusters)

While CAM provides two clustering methods in the earlier package (KMC and APC), K-means clustering is usually the first option. The reason is that when data contains thousands of genes (features) with dozens of samples, the computation time of Affinity Propagation Clustering (APC) is not feasible. However, as we know, there are some disadvantages of K-means clustering. For example, K-means clustering needs initialization, and which does not guarantee a global optimum solution (MacQueen, 1967). That is, if K-means clustering may give different results with different initializations. Since the clusters from K-means clustering are for simplex vertex identification in CAM, CAM may produce unstable deconvolution results. Also, the reason CAM needs a clustering method for pre-processing is to suppress the noise before finding the convex hull. However, since we cannot control the size of each cluster from K-means clustering, the noise may be suppressed at varying degrees in different clusters, which may distort the shape of the convex hull. Though we can change the number of clusters to affect the size of clusters in K-means, it is not guaranteed that the size of all clusters will change with the number of clusters, and the size of all clusters may still not be the same usually. Also, it is well-known that K-means clustering is sensitive to the outliers, so if there are many outliers, K-means clustering is not an ideal choice.

To address the problems of K-means clustering, we prefer a method which can control the size of each cluster to a maximum specified radius, where the radius threshold can be considered as a parameter. Ideally, it should be a deterministic method so there is no randomness in the result, and as robust as possible to outliers. Accordingly, here we propose an alternate clustering method called Radius-Fixed Clustering (RFC). The idea of radius-fixed clustering is that first we need to decide the value of the radius threshold, and then iteratively remove the clusters satisfying some requirements one by one from the dataset until there is no clusters which meet the requirements. The threshold here we selected is cosine similarity, since Euclidian distance is distorted after projection, but cosine similarity will not change. The steps of radius-fixed clustering are as following (**Fig. 1A**):

1. Set a radius (though we choose cosine similarity for CAM; in other applications different distance or similarity measures are acceptable).
2. For each gene (point), compute how many genes are within the radius. That is, set each gene as the center of a hyper ball, the radius of which is the one set in step 1, and then compute how many genes are within the hyper ball.
3. Find out the hyper ball with the greatest number of genes inside, and then remove all the genes in the hyper ball from the data.
4. Repeat Step 2 and 3 until there is no gene left or the number of genes in the hyper balls are below a certain value.
5. All the hyper balls removed from the data are the clustering results by radius-fixed clustering.

Since we have set the “radius” of each cluster at the beginning, it is guaranteed that no cluster would be larger than the radius. Thus, we can control the size of each cluster efficiently, or at least we can make sure that when computing the mean of each cluster, there is no gene which is far from the center of the cluster. Also, if we set a threshold for the number of genes in each cluster, there may be some genes which do not belong to any cluster. Since the target of the clustering step is to suppress the noise, it is acceptable that if there are some genes which are not clustered. That is, the target of radius-fixed clustering is to “down-sample” the simplex for de-noising, not for “clustering” all the genes to clusters. Also, if these “not clustered” genes are outliers, actually it would even make our de-noising more accurate. Moreover, in biology, genes always work together (pathways) (Kelley and Ideker, 2005). That is, a bunch of genes will have similar patterns since they have related functions. Thus, when using radius-fixed clustering, setting a threshold for member number of a cluster can not only remove the outliers, but also follow the biology principle. See below, illustration of radius-fixed clustering. (a) After deciding the radius (the radius of the red circle), we can set each gene as center, and then find which circle contains the greatest number of gene (red circle, seven points). (b) Since the points in the red circle in (a) are removed, the next one contains four. (c) The next one contains three points. (d) The final one contains two, if we set all the clusters should contain at least two points.


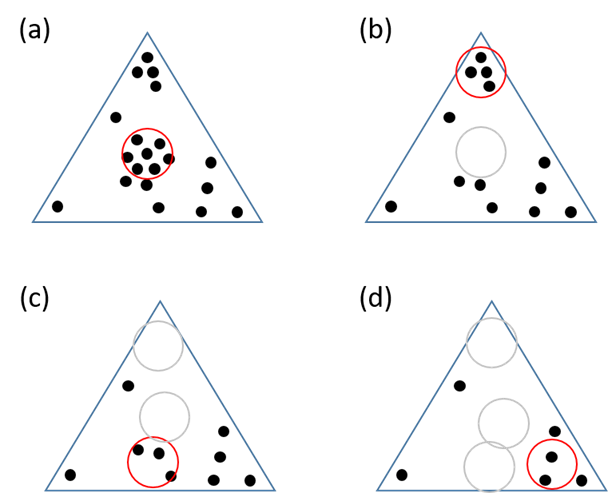


We applied RFC to a real gene expression dataset (3 cell types, 30,000 genes). The experimental results show that RFC performs comparatively as KMC yet with higher tightness and density, unique solution, reduced outlier contamination, and proper cluster numbers (Left: KMC; Right: RFC).


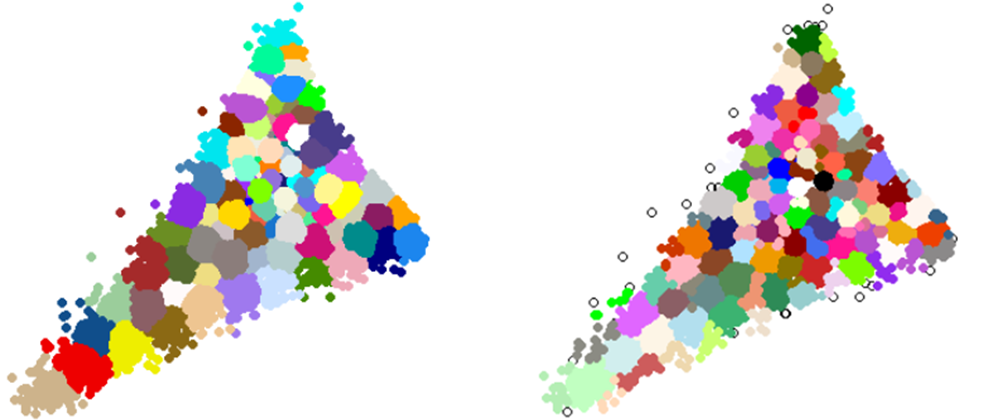


**Convex hull identification**

The core idea of CAM is to identify the clustered marker genes for different cell types, which are located on the vertices of the scatter simplex. However, it is hard to find the optimal simplex directly, so CAM finds the initial convex hull of the data first. That is, the optimal simplex should be a subset of the complete convex hull. Currently, the most popular method is Quickhull (Barber, et al., 1996), a divide and conquer approach, the idea of which is similar to quicksort. Essentially, Quickhull finds a convex set and remove the internal points iteratively, until no points remain to be removed. With respect to the number *N* of features for two or three dimensions, the computational complexity of Quickhull is about $O(N\log N)$. For a *d*-dimensional case (i.e., *d* samples or mixtures), the computational complexity becomes $O( {N^{\frac{d}{2}}}/{\left( \frac{d}{2} \right)!} )$.

Recall that the objective is to find the extreme points in the data. Given the definition of a convex set, any point $p_{i}$ in the data can be the convex combination of all points:

$$\begin{aligned} \sum_{i=1}^{N} \lambda_{i}p_{i} : \lambda_{i}\geq0 for all i, and \sum_{i=1}^{N} \lambda_{i}=1.\#\left( 3 \right) \end{aligned}$$

However, the definition of the extreme point is that the point can be constructed by just itself (the coefficients of the other points are all zero). That is, we are finding some points the linear combination of which are only themselves with some constraints on the coefficients. This problem can be formulated as a linear programming problem (Pardalos, et al., 1995),

$$\begin{aligned} \min\lambda_{j} s.t. \sum_{i=1}^{N} \lambda_{i}p_{i}=p_{j}, \sum_{i=1}^{N} \lambda_{i}=1, \lambda_{i}\geq0 for all i.\#\left( 4 \right) \end{aligned}$$

We can test all $p_{j}$ in the equation 4.6, and if $\lambda_{j}$ is not zero, then the corresponding $p_{j}$ is an extreme point. There are several advantages to using linear programming to find the convex hull. First, though the time complexity of linear programming is just weak polynomial, it is faster than the one of Quickhull with respect to simplex dimension (**Table S1**). Second, the number of clusters in CAM is usually around 100, and all clusters can be tested simultaneously. With linear programming, we can reduce computational time using parallel computing. Third, if we “relax” the convex hull, for example, the points near the boundary could be also identified as the extreme points, we can manipulate the constraints of the linear programming.

**Table S1**. Comparison of the computational time for identifying the initial scatter simplex, required by Quickhull versus LP, tested in the simulation data.


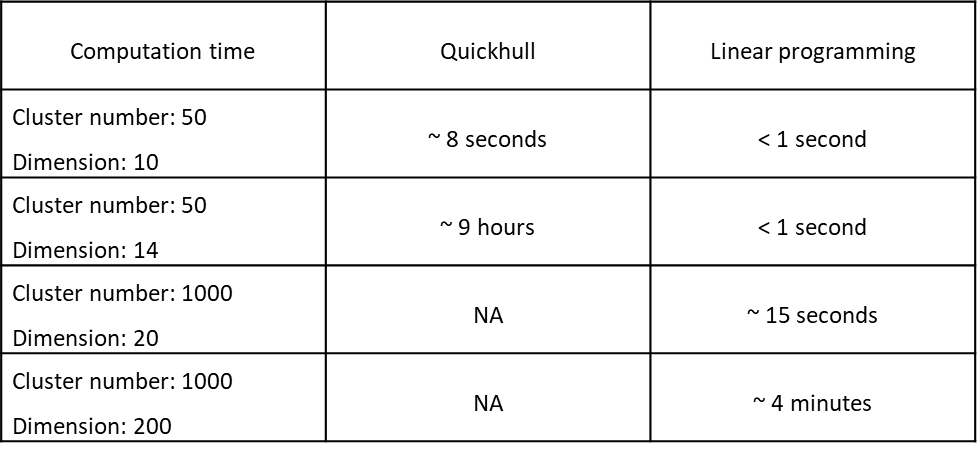


**Identification of the optimal simplex**

Given the number of sources *K*, finding the optimal simplex from the convex hull is a combinatorial search problem. That is, there are $C_{K}^{Q}$ combinations to form a (*K*-1)-simplex. To reduce the computational complexity of this combinatorial search, we propose to directly minimize the reconstruction error via the strategy of combined Sequential Forward Floating Search (SFFS) and Sequential Backward Floating Search (SBFS) (Pudil, et al., 1994). More importantly, in such a greedy search, SFFS or SBFS searches different values of *K* and the optimal simplex, simultaneously, reducing the computational time significantly. The detailed steps of SFFS are as follows (SBFS can be similarly designed):

1. Test all *Q* candidates, and select the one with lowest reconstruction error with $\mathbf{X}$ by NNLS. Then move the selected one from the candidates to the feature set.
2. Select one from the remaining candidates, and use this one and the feature set to compute the reconstruction error. After testing everyone in the remaining candidates, move the one with the lowest reconstruction error to the feature set. Record the reconstruction error for the current number of features in the feature set.
3. Remove one from the feature set temporarily, compute the reconstruction error for the current feature set, and then move the one back. After testing everyone in the feature set, if the lowest reconstruction error is lower than the recorded for the current number minus one of features in the feature set, remove the corresponding feature and replace the recorded reconstruction error.
4. Repeat Step 3 until no lower reconstruction error can be found.
5. Repeat Step 2 – 4 until there is no candidate left.

Because both SFFS and SBFS will test all possible values of *K* within their loops, we can compare the reconstruction error from SFFS and SBFS for the same source number, and pick the one with lower reconstruction error as the final result for certain source number (**Table S2**).

**Table S2**. Comparison of the computational time for identifying the optimum scatter simplex, required by Margin of error versus smart floating search, tested in the simulation data.


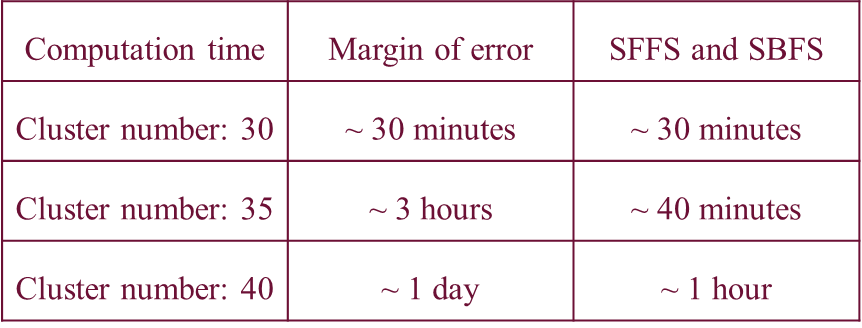


To further reduce the computational complexity, we also approximate the reconstruction error using the lowest “unexplainable portion” (relative to all others) when deciding which candidates should be removed from the feature set. The unexplainable portion of ***X*** is the residue multiplying the corresponding row in the S matrix for each of the feature set fitted via NNLS. Estimating unexplainable portions is efficient because only the selected feature of the feature set needs to be fitted, not the entire $\boldsymbol{X}$. See below, Illustration of SBFS with reconstruction error and unexplainable portion with four features in the feature set is shown below. (a) The reconstruction error the sum of the square of the residue by NNLS fitting. (b) In the Step 2 of SBFS, NNLS for fitting ***X*** is performed for excluding each feature in the feature set (four times in this figure) to compute the reconstruction error. (c) While we still need to perform NNLS four times, we can fit a matrix with only one column, not the whole ***X***, so the computation time decreases significantly.


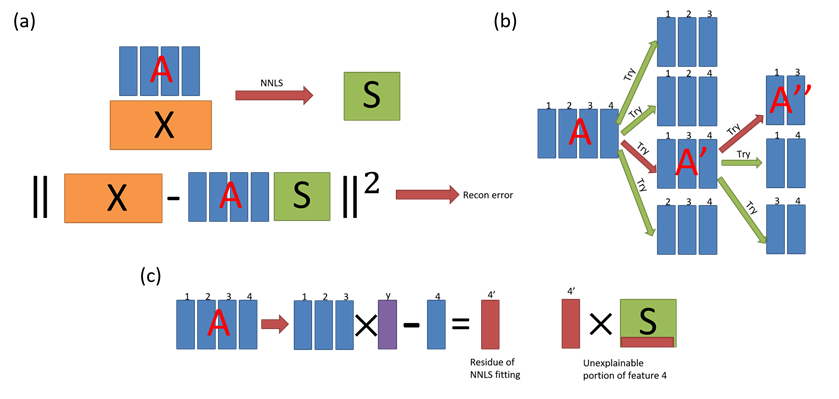


**Performance index** (multiple quantitative measures)

Four quantitative measures are used to evaluate estimation accuracy, namely Root Mean Square Error (RMSE), average cosine score, source-wise proportion correlation, and sample-wise proportion correlation. Specifically, RMSE is given by (Oba, et al., 2003; Stekhoven and Bühlmann, 2012)

$RMSE= \sqrt{\frac{\sum_{\Omega} {(\hat{A}-A)}^{2}}{|A|}}$,

where $A$ is the ground truth proportion matrix, $|A|$ is the total number of entries, and $\hat{A}$ is the estimated proportion matrix.

## Results (with supplementary figures and tables)

We have developed the open-source Bioconductor R package that implements and tests the earlier versions of the CAM based deconvolution tool (Chen, et al., 2020), already freely available at <http://bioconductor.org/packages/debCAM>. The experimental evaluation and comparison of CAM method with the most relevant peer methods have been previously reported (Chan, et al., 2008; Chen, et al., 2022; Chen, et al., 2020; Wang, et al., 2010; Wang, et al., 2016; Zhu, et al., 2016). We have developed and submitted the open-source Bioconductor R package for CAM3.0, with the prototype version freely available at <https://github.com/ChiungTingWu/CAM3/>, (https://github.com/Bioconductor/Contributions/issues/3205)

**Muscle regeneration**

This case study aims to investigate whether and how the interactions among the molecular distinctive cell types may affect the normal or failed (muscular dystrophies) muscle regeneration. Because muscular dystrophy remodeling is expected to share many features with normal muscle regeneration (**Fig. S5**), we sought to determine the molecular mechanisms underlying observed failure of muscle regeneration in dystrophin-deficient muscle through hypothesis generation using muscle mRNA profiling data (Bakay, et al., 2006; Dadgar, et al., 2014).


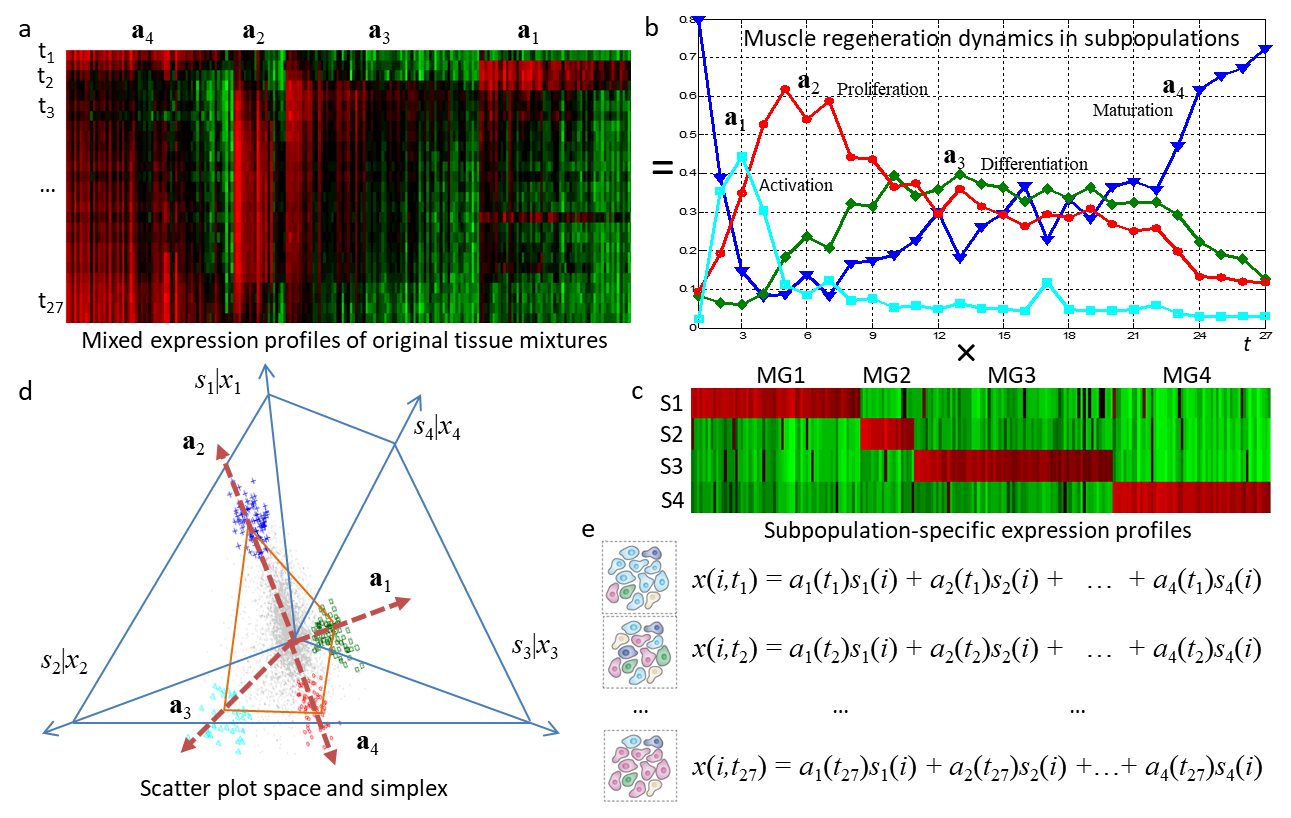


**Figure S5**. CAM framework for unsupervised *in silico* deconvolution of tissue heterogeneity that uncovers *de novo* subpopulations and repopulation dynamics. In CAM model, *s_k_*(*i*) is the expression level of gene *i* in subpopulation *k*, *x_j_*(*i*) is the expression level of gene *i* in heterogeneous sample *j*, and *a_jk_* is the proportion of subpopulation *k* in heterogeneous sample *j*. In our mathematical modeling of transcriptional heterogeneity, mRNA expression levels from a mixture of multiple subpopulations are modeled as the weighted average of the expression levels from a set of distinct subpopulations, where the weights describe the proportions of each distinct subpopulation in the overall population. (**a**) Heatmap of mixed gene expression time-course from heterogeneous muscle tissues sampled across 27 time points during normal muscle regeneration, plotted over 214 CAM-detected subpopulation-specific marker genes whose expressions are exclusively enriched in a particular subpopulation. (**b**) Repopulation dynamics of the four distinct subpopulations/phases (Activation, Proliferation, Differentiation, and Maturation) in terms of relative individual proportions estimated by CAM. (**c**) Heatmap of marker gene expression levels in the four distinct pure subpopulations/phases estimated by CAM. (**d**) Geometry of the mixing operation in scatter space that produces a compressed and rotated scatter simplex of original mixed gene expressions whose vertices host subpopulation-specific marker genes and correspond to mixing proportion vectors. (**e**) Mathematical description on the mixed gene expression readout from heterogeneous mixtures of multiple distinct subpopulations.

Muscle regeneration involves highly synchronized activations of various cellular processes, and here we ask whether an unsupervised deconvolution of bulk tissue gene expression data is able to discern the molecular cell types and proportional dynamics. We applied CAM to a time-course gene expression dataset obtained from a mouse muscle regeneration process (GSE469). The time-course gene expression data were acquired over 27 successive time points after the injection of cardiotoxin into the mouse muscle, which induces staged muscle regeneration (**Fig. S5a**). The MDL criterion suggests *K* = 4 as the number of molecularly distinct cell types indicated by the four vertices of scatter simplex (**Fig. S5d**), and CAM uncovers the temporal repopulation dynamics of the four *de novo* subtypes at each time point (**Fig. S5b**), over 214 subtype-specific markers (**Fig. S5c**). Using gene set enrichment analysis, these subtypes are found to be closely associated with the activation, proliferation, differentiation, and maturation of muscle regeneration.

By a closer look into the cell type specific gene expression profiles estimated by CAM in a separate study on muscular dystrophies (Dadgar, et al., 2014), we found that TGFβ-centered networks strongly associated with pathological fibrosis and failed regeneration were also induced during normal regeneration, but at distinct time points (temporal parsing into sub-networks). We hypothesized that asynchronously regenerating microenvironments was the underlying driver for fibrosis and failed regeneration. We validated this hypothesis using an experimental model of focal asynchronous bouts of muscle regeneration in wild-type mice. Laser capture microdissection and mRNA profiling of each notexin-injection site, and the in-between area was done, showing reproducibly different genome-wide microenvironment data (Dadgar, et al., 2014). Using CAM based tissue deconvolution results and large human biopsy data sets, we have developed a novel model of the gradual failure of regeneration as a function of age/time in the muscular dystrophies, with experimental evidence in mouse models, where asynchronous remodeling puts microenvironments into a ‘arrested development’, unable to progress normally through the time-dependent regeneration process (Dadgar, et al., 2014). This model will have broad implications for many chronic inflammatory states.

**Human brain lifespan**

We first re-validated CAM based deconvolution on the benchmark mouse brain data involving neuron, astrocytes, and oligodendrocytes cell types (GSE19380) (Kuhn, et al., 2011). The individual gene expression profiles of the three primary subtypes were variably and experimentally mixed, where the cells were cultured and the mRNAs were extracted separately. Without using any prior information, CAM accurately detected the number of subtypes, identified subtype-specific markers, and estimated the proportions and subtype-specific gene expression profiles. These results were assessed against the ground truth (Wang, et al., 2016). Importantly, CAM successfully detects not only the majority of the known but also many de novo marker genes associated with brain cell types (**Fig. S6a**).


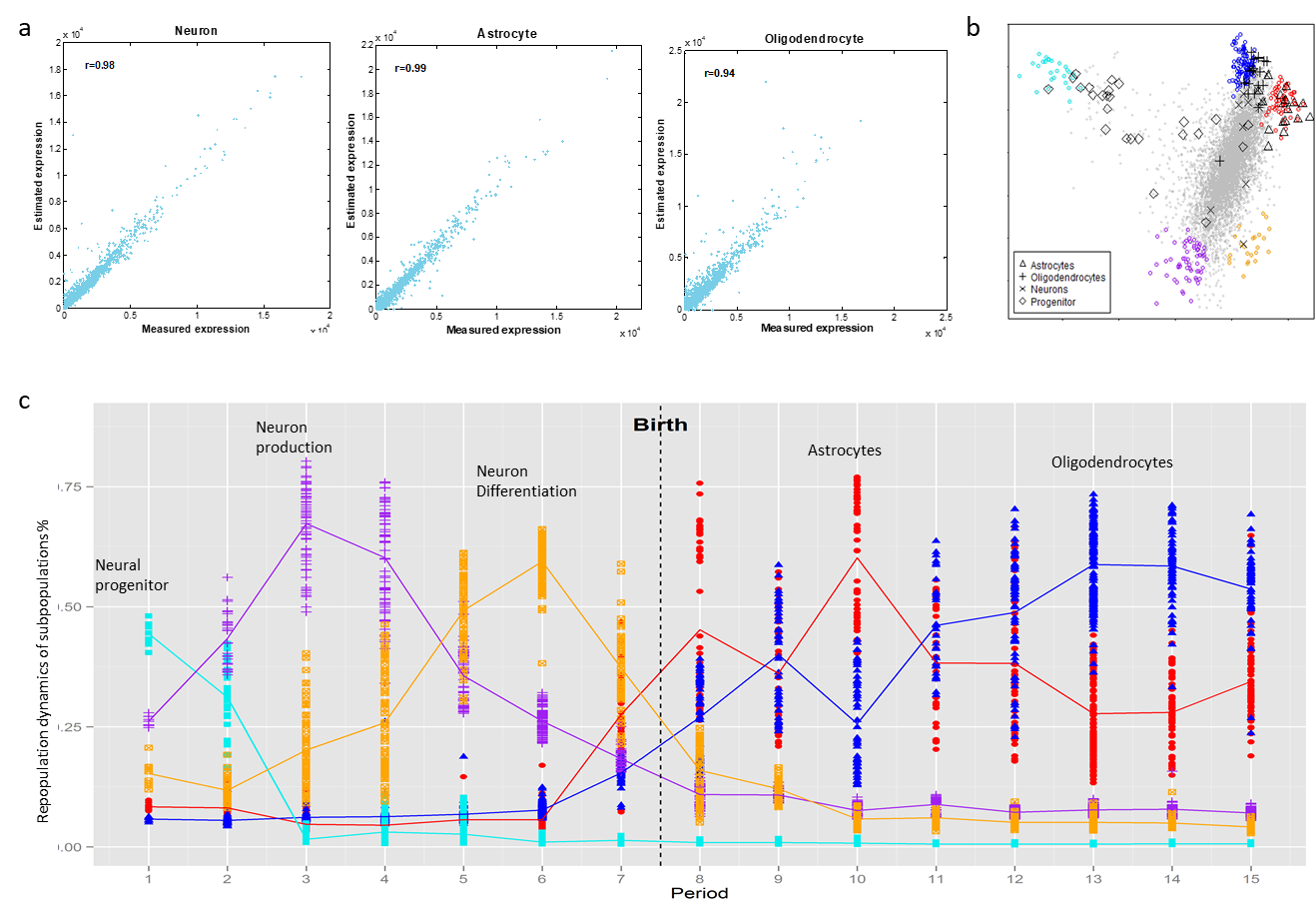


**Figure S6**. Validation and application of CAM in identifying *de novo* subpopulation-specific marker genes and deconvoluting mixed gene expression profiles, with distinct subpopulations of progenitor, neuron, astrocyte, and oligodendrocyte, at their various development states. (**a**) Scatter plots of the estimated subpopulation-specific gene expression profiles deconvoluted from heterogeneous tissue samples (GSE19380) by CAM, plotted against the measured gene expression profile from pure subpopulations, with an almost perfect correlation coefficient. (**b**) Identification of marker genes by CAM from the simplex of mixed gene expression data (HBT GSE25219) (Gray dots: all genes forming scatter simplex. Light-blue dots: marker genes of neural progenitor. Magenta dots: marker genes of neuron in production. Brown dots: marker genes of neuron in differentiation. Red dots: marker genes of astrocyte. Blue dots: marker genes of oligodendrocyte. Gray symbols: *a priori* marker genes for four known cell subpopulations.) (**c**) Based on the gene expression time-courses of cell subpopulations in human brain sampled at various developmental periods, CAM detected *de novo* subpopulation/state-specific marker genes and dissected mixed gene expression profiles into subpopulation/state-specific gene expression patterns. The result revealed repopulation dynamics of five distinct subpopulations/states that were undetectable by either global profiling or supervised deconvolution. The relative proportions of these subpopulations or states estimated by CAM, plotted as a function of time, match well with the previously-validated cellular repopulation dynamics during brain development.

We then applied CAM to the benchmark human brain lifespan data set, Human Brain Transcriptome (HBT) (GSE25219, Illumina Human 49K Oligo array, 923 samples with 17,565 probes) (Kang, et al., 2011). Using the time-courses of the bulk gene expression profiles sampled at various human brain developmental periods and lifespans, CAM first detect *de novo* subtype-specific markers (**Fig. S6b)**, i.e., the vertex-residing markers detected by CAM directly from the simplex of bulk gene expression data. These blindly detected markers match well with the *a priori* markers associated with the cell subtypes in the human brain (**Table S3**). Based on the expression patterns of these blindly detected markers, CAM then reconstructs the temporal repopulation dynamics of five molecularly distinct subtypes or cell states that were undetectable by either global profiling or supervised deconvolution (**Fig. S6c**). We also separately applied CAM to (four different regions) region-specific HBT data sets of the bulk tissues sampled from the four different cortex regions (**Figs. S7**). The obtained region-specific temporal repopulation dynamics are similar to those estimated from the whole data set (**Fig. 1D**). We further replicated the HBT deconvolution results in an independent benchmark data set, Braincloud (GSE30272, Affymetrix Human Exon 1.0 ST Array, 269 samples with 30,176 probes) (Colantuoni, et al., 2011). The deconvolution result in terms of brain cell repopulation dynamics is consistent with HBT (**Fig. S8**). Again, the blindly detected markers in this replication study match well with the *a priori* markers associated with the cell types in the human brain (**Table S4**).

**Table S3**. Comparative counts of *a priori* markers enriched in CAM3.0-identified cell types (HBT: * count of probes measured in GSE25219 and linked to a priori marker genes).


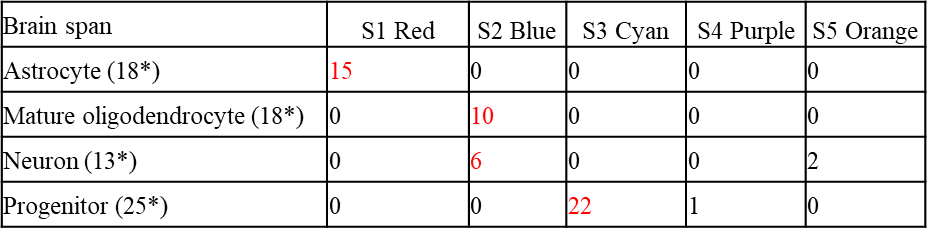


**Table S4**. Comparative counts of *a priori* markers enriched in CAM3.0-identified cell types (Braincloud: * count of probes measured in GSE30272 and linked to a priori marker genes).


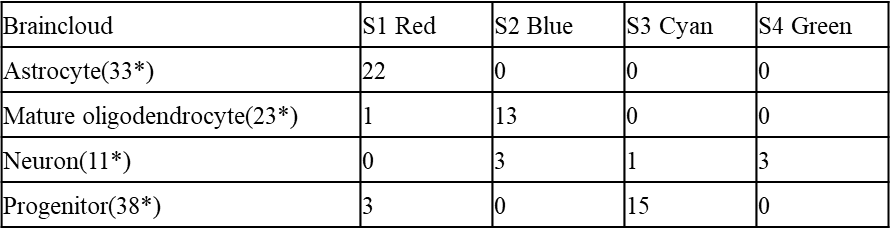


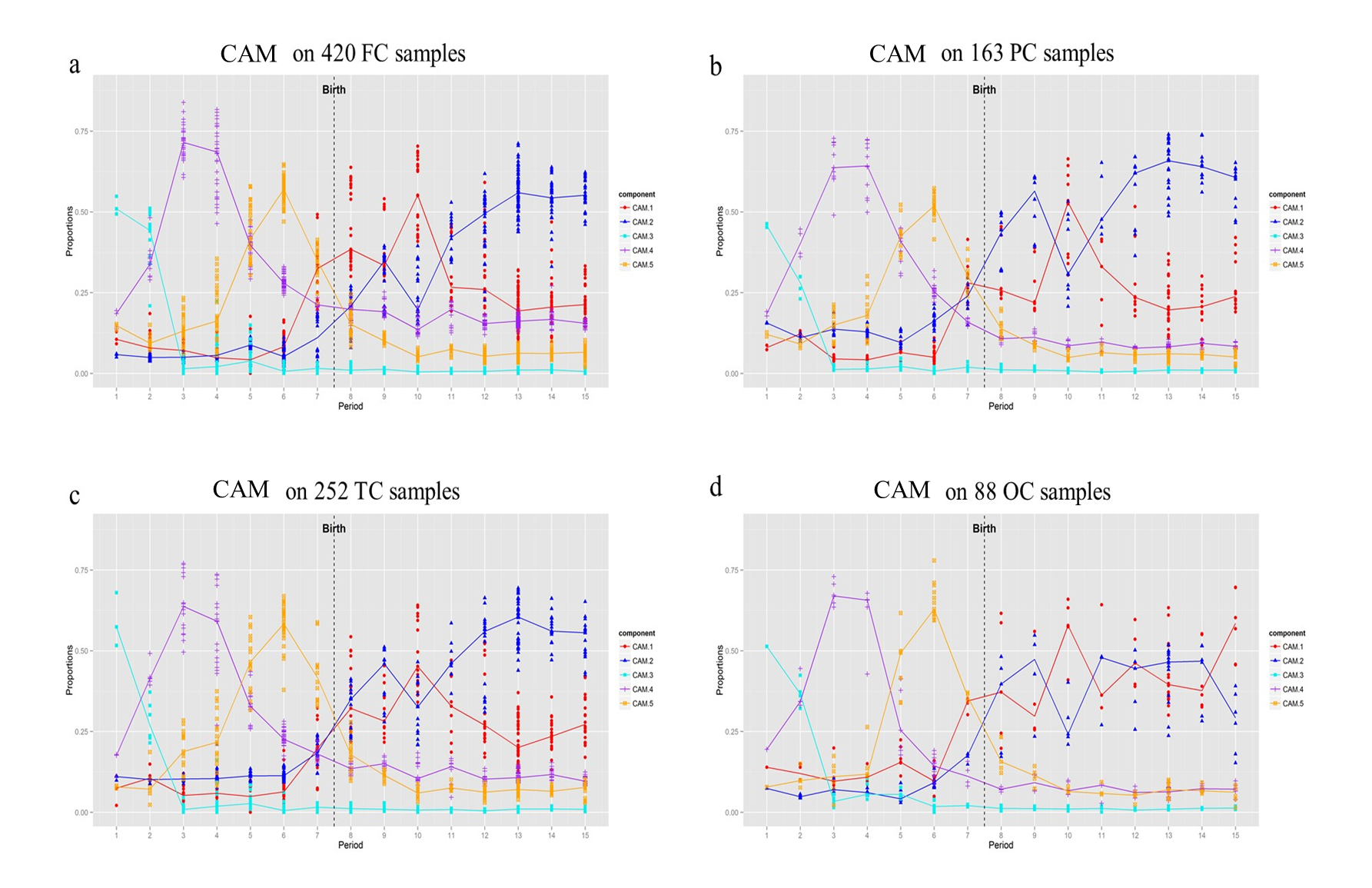


**Figure S7**. The Repopulation dynamics of distinctive subtypes/states during life span in each of four cortices estimated by CAM applied to gene expression data from HBT cortex tissues.


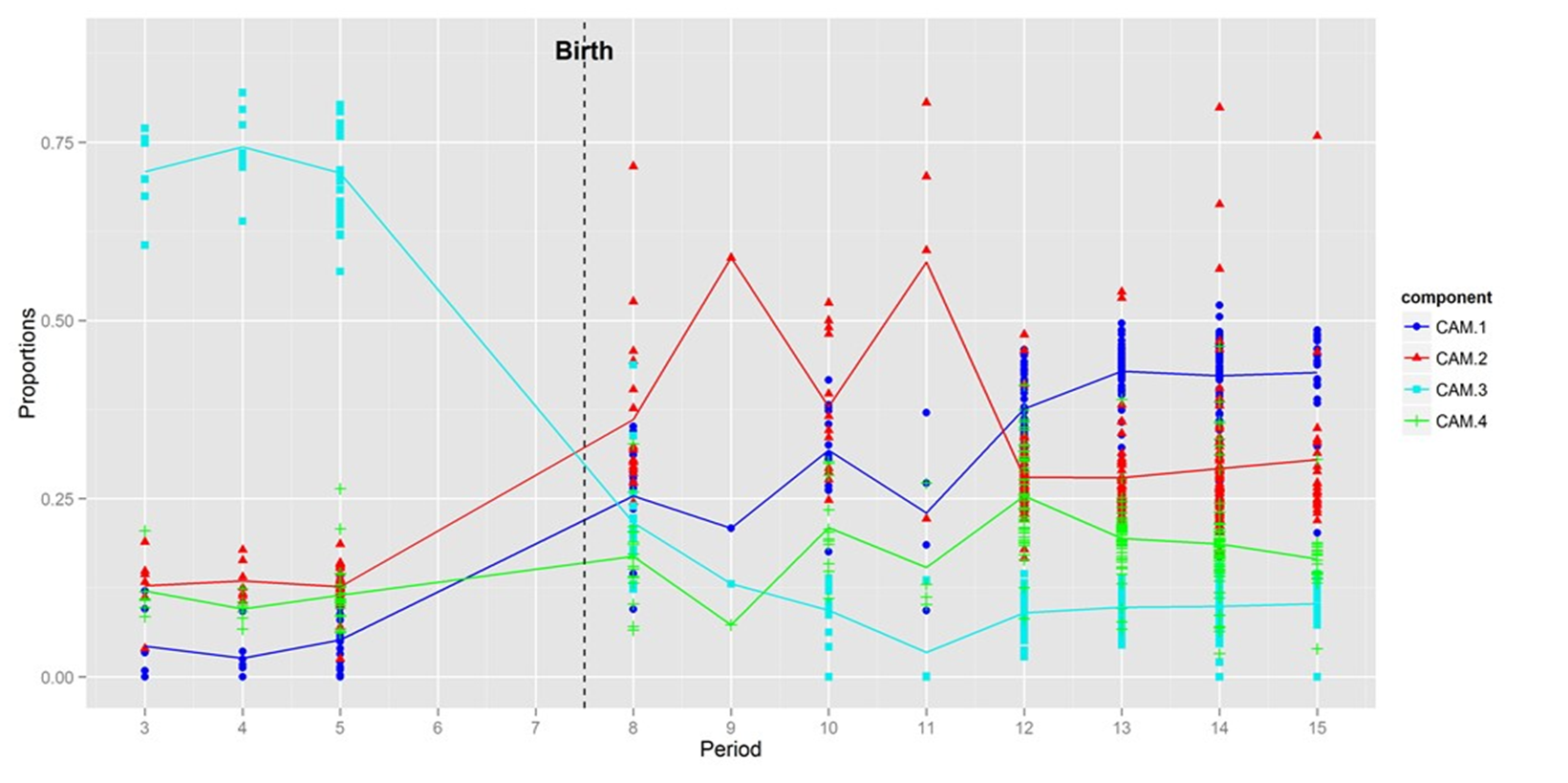


**Figure S8**. Application of CAM to deconvolve Braincloud data (GSE30272) that replicate the repopulation dynamics of progenitor, neuron, astrocyte, and oligodendrocyte, at their various development states, consistent with what obtained from HBT data. The Repopulation dynamics of distinctive subtypes/states during life span estimated by CAM applied to gene expression data from Braincloud cortex tissue samples.

The relative proportions of these primary brain cell subtypes, blindly estimated by CAM and plotted as a function of time, match well with the previously-validated repopulation dynamics data about human brain development and lifespan (Stiles and Jernigan, 2010). Neuron production in humans begins on embryonic day 42, extends in most brain areas, and is largely complete by midgestation. Among the stem cell lines that emerge during gastrulation are the neural stem cells. The neural stem cells are capable of producing all of the different cells that make up the brain and central nervous system, and for this reason the neural stem cells are usually called the neural progenitor cells (Stiles and Jernigan, 2010). While most neurodevelopmental events involve the proliferation of neural elements, two important processes involve substantial loss of neural elements. These two processes include naturally occurring cell death, which involves the normal loss of 50% or more of the neurons within a brain region; followed by the systematic elimination of up to 50% of between-neuron connections. cascade. Apoptosis has been documented within all of the neuronal and neural progenitor cell compartments in the human brain. Across the cortex, rates of apoptosis within all layers is high, reaching 70% in some regions. Importantly there is strong evidence of high levels of cell death in the neural progenitor population. population. In contrast, proliferation of glial progenitors, while beginning prenatally, continue for an extended protracted period after birth as oligodendrocytes and astrocytes differentiate. In summary, brain development involves overproduction of neurons and glial cells, followed by various regressive events.

**In-house brain bulk data**

Brain is made up of hundreds of different cell types. While the major classifications are neuronal cells and glial cells, each of these has many subcategories based on their morphology or functions (Mancarci, et al., 2017). Our in-house brain data were gene expression profiles measured from 129 parietal cortex (PC) tissues and 129 cerebellum (CB) tissues. The major cell types in PC and CB regions are shown in **Table S5**, with *a priori* markers available from the literatures (Xu, et al., 2013). CAM3.0 identified three and four subtypes, respectively. The enrichment of *a priori* markers in each deconvoluted subtype indicates CAM3.0 successfully finds three major brain cell types – astrocyte, mature oligodendrocyte, and neuron – in the human parietal cortex (**Fig. S9a**). Among much more complex compositions in the cerebellum, CAM3.0 also distinguish four major cell types (**Fig. S9b**). CAM3.0-identified markers located in simplex vertices match well with part of *a priori* markers (**Fig. S9, Tables S6-S7**). Two simplex scatter plots reflect astrocyte and mature oligodendrocyte, as two major glia cell types, have distinct patterns at molecular expression level from others in both cortex and cerebellum. The CAM3.0-estimated proportions of cell types (**Fig. S10**) indicate neuron cell types account for a larger proportion than the sum of two major glia cell types.

**Table S5**. Cell types (counts of *a priori* markers) in brain tissues detected by (Xu, et al., 2013).

| 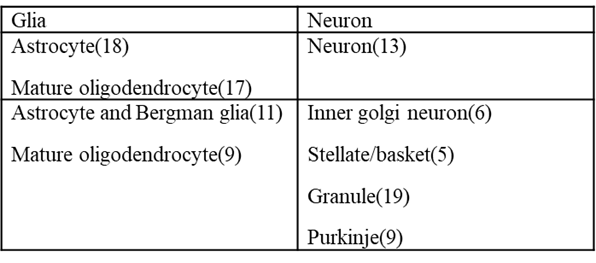  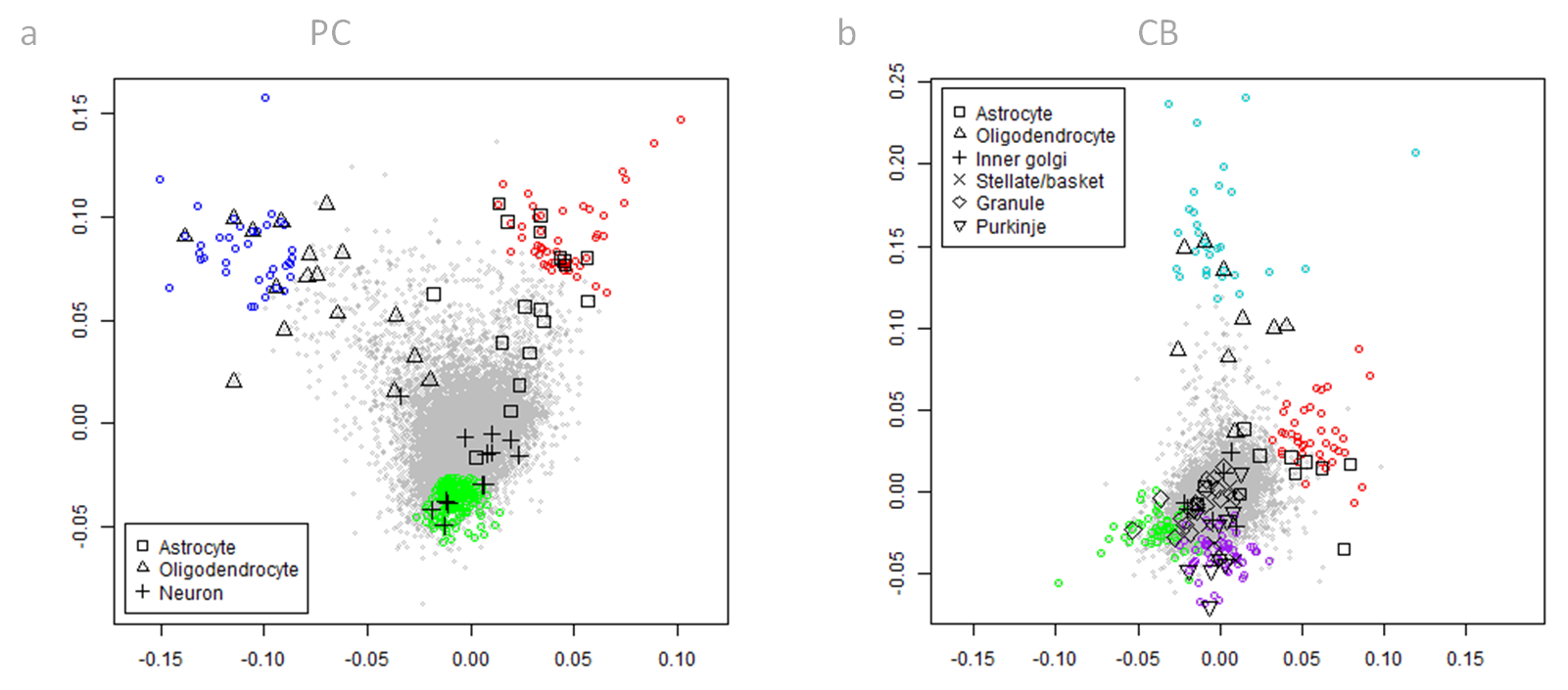 |
| --- |

**Figure S9.** Scatter simplex of complex tissues from parietal cortex and cerebellum (Gray dots - all genes forming scatter simplex; colored dots – markers identified by CAM3.0; black symbols - a priori markers for known cell types).

**Table S6**. Counts of *a priori* markers enriched in CAM3.0-identified cell types (PC).


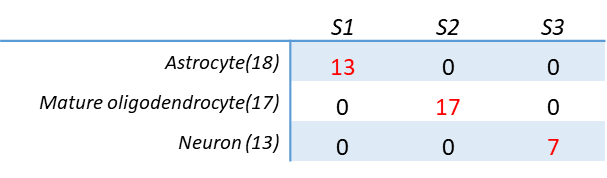


**Table S7**. Counts of *a priori* markers enriched in CAM3.0-identified cell types (CB).


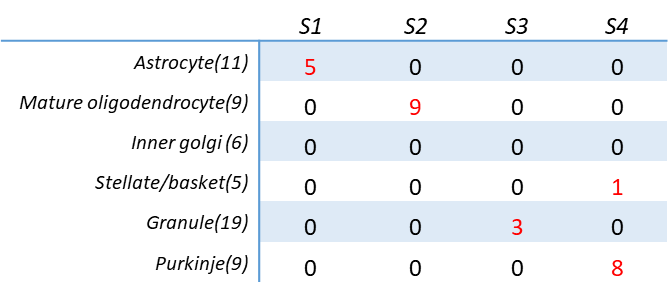


| 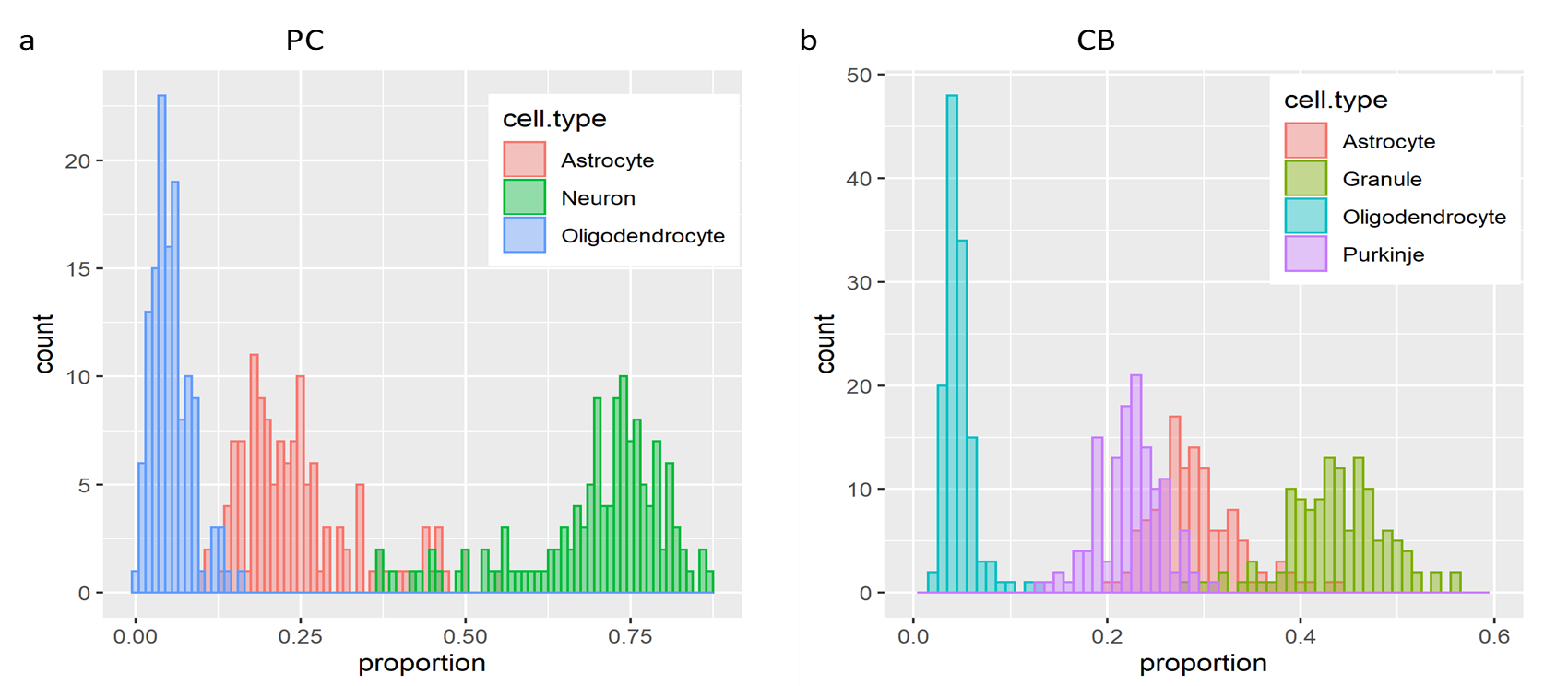 |
| --- |

**Figure S10.** Histogram of estimated proportions of CAM3.0-identified cell types in parietal cortex and cerebellum (implemented in the prototype software CAM).

While the two major types of cells in the brain are known to be glia and neuron, the true ratio of glia to neurons in the brain remains a mystery. One of the recent studies using an efficient cell counting method provides compelling evidence for 1:1 ratio on four whole human brains (Azevedo, et al., 2009). The same study also reveals that the ratio of glia to neurons in the brain varies from one region to another, sometimes dramatically, e.g., 3.76:1 in the cerebral cortex versus 1:4.3 in the cerebellum (Azevedo, et al., 2013; Azevedo, et al., 2009). However, other scientists have argued that more rigorous studies are needed in which just about every known or unknown marker for both neurons and glia is used to capture as many of the different cell types as possible.

**Vascular bulk tissue proteomics data**

At the molecular level, atherosclerosis can be defined as an assembly of hundreds of intra- and extra-cellular proteins that jointly alter cellular processes and produce characteristic remodeling of the local vascular environment. Ultimately, these proteomic changes produce the lesions responsible for most ischemic cardiovascular events. The ability to identify marker proteins characteristic of early- and late-stage pathological tissue states would have meaningful clinical impact, but challenged by the heterogeneous nature of whole tissue proteomic profiling involving varying proportions of different tissue phenotypes.


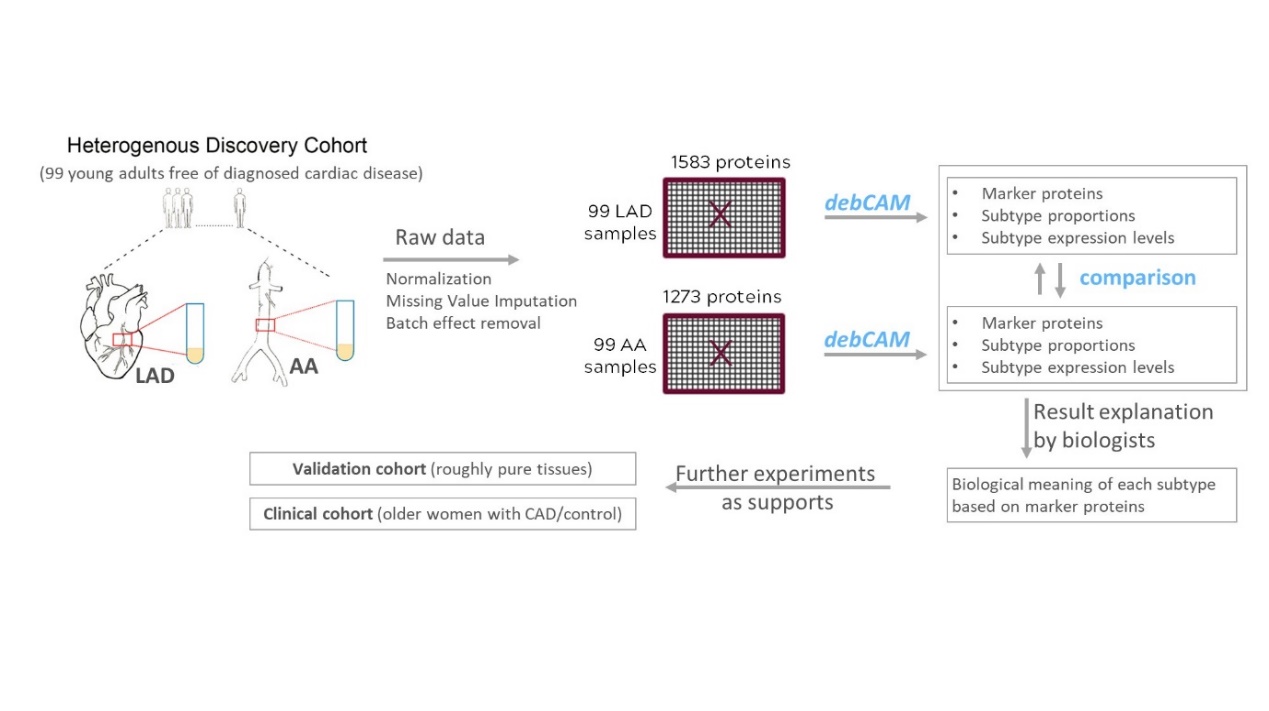


**Figure S11**. CAM3.0 analysis workflow (implemented in the prototype software debCAM) for proteomics data acquired from two vascular regions (LAD and AA). CAM3.0 results are cross-validated by comparing marker proteins detected in two regions, interpreted by biologists and further supported by biological validation cohort and clinical cohort.

In one of our projects for Global Analysis of Coronary and Abdominal Aortic Proteomes, specimens were collected from left anterior descending coronary artery (LAD) and abdominal aorta (AA) regions in 99 donors free of clinical/diagnosed cardiovascular disease (**Fig. S11**). A trained pathologist scored each specimen for surface involvement of fatty streak (FS), fibrous plaque (FP) and normal (NL). 1583 proteins in LAD and 1273 proteins in AA are quantified by the DIA-MS technique with less than 50% missingness. After data normalization and imputation, CAM3.0 algorithm was performed and the results indicated four and two distinct expression subtypes in the LAD and AA specimen, respectively (**Fig. S12**). We also applied a supervised method called csSAM (Shen-Orr, et al., 2010), which deconvolute $\mathbf{S}$ from $\mathbf{X}$ with prior $\mathbf{A}$, to estimate tissue subtype-specific expression profiles by pathologist-scored proportions of three tissue subtypes and further obtained associated markers using OVE-FC scheme. Although subtype proportions observed by the pathologist were quite crude, the overlap of CAM3.0-identified markers and csSAM-identified markers (**Tables S8** and **S9**, numbers in parentheses are counts of markers) provided clues about the biological interpretation of CAM3.0-identified subtypes in LAD/AA. While FS and NL subtype in AA were not separated by CAM3.0 due to insufficient mixture diversity, LAD detected an extra subtype (CAM3.0-NL1), which may reflect complex compositions of normal tissues.


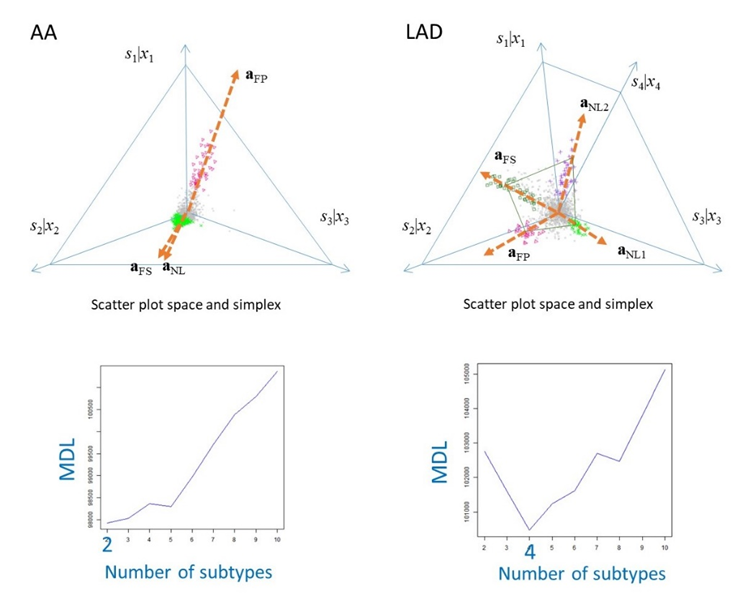


**Figure S12**. The scatter simplex and MDL curve associated with AA and LAD samples.

As pathologist-scored proportions were just visual inspection of the arterial samples and thus, less reliable than CAM3.0 estimations, our collaborator biologists started focusing on CAM3.0-identified subtypes, especially FP subtype whose abundance represents the vascular FP burden and indicates more severe status than NL and FS. Biologists searched the literatures and public database to confirm that CAM3.0-identified FP markers are associated with lesion/FP. Besides, the 34 FP markers blindly detected from LAD and 49 FP markers from AA have an overlap of 21(33.9%), cross-validating the unsupervised discovery from each other.

**Table S8**. Counts of marker proteins detected in LAD by csSAM and CAM3.0 and their overlaps.


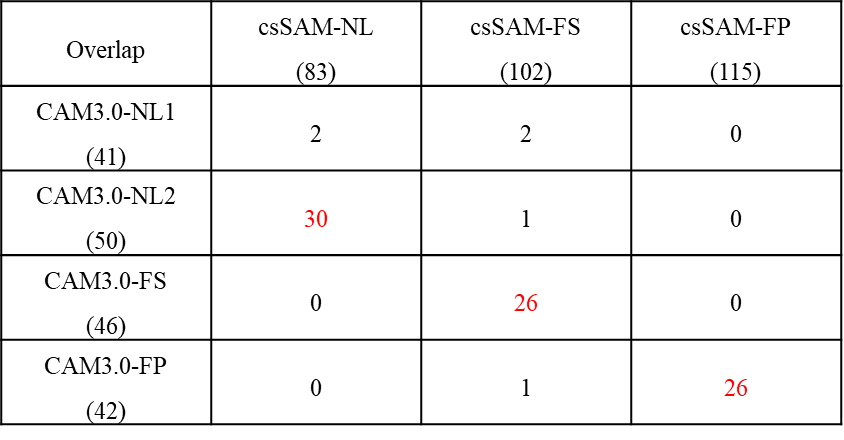


**Table S9**. Counts of marker proteins detected in AA by csSAM and CAM3.0 and their overlaps.


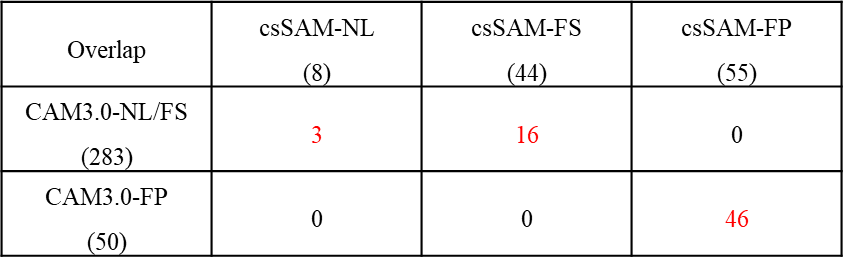


As the enrichment levels of FP markers will be good candidates for representing the vascular FP burden and marking early atherosclerosis, biologists conduct two more experiments to further support putative FP markers from CAM3.0. The first validation cohort collected a set of specimens isolated from an orthogonal, separate cohort of donors (Parker, et al., 2020). These pure aortic tissues are isolated from regions with no pathology (pure normal, n=3) and regions completely comprised of fatty streak (pure FS, n=3) or fibrous plaque (pure FP, n=4) luminal surface involvement. 1478 proteins were quantified from the pure specimens by the same DIA-MS technique as LAD/AA tissue profiling used so that expression levels could be comparable from two independent experiments. 58 of 62 putative FP marker proteins are quantified in a validation cohort and most are enriched in pure FP specimens compared to pure FS and pure normal specimens. Statistical comparison (OVESEG-test (Chen, et al., 2019)) of the expression profiles among pure FP, pure FS and pure normal tissue specimen shows 18 of 58 putative markers have a significant enrichment with q-value < 0.02. The expression values of FP-marker estimated from either LAD or AA correlated well with those measured from pure specimens, with correlation coefficients of 0.661~0.919 (**Table S10**). The second validation cohort collected clinical data to examine putative FP markers’ performance in classifying coronary artery disease (CAD) patients and healthy women. High intercorrelation was observed among FP marker proteins. Thus, we applied elastic net variable selection within a logistic regression analysis to select a panel of 10 proteins that achieved high AUC and low misclassification rate (Parker, et al., 2020).

**Table S10**. Correlation coefficient between CAM3.0-estimation and pure measurement over FP signature genes (a more stringent setting w/o many less-differentially expressed genes).


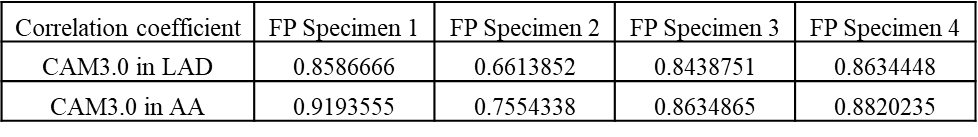


## Experimental comparison with peer methods

In relation to existing deconvolution software tools, we conducted both functional and experimental comparisons between CAM3.0 and the most relevant peer methods. Table S11 summarizes the functional comparison, concerning the methods’ functionalities, requirements, and methodological principles. As a fully unsupervised method, CAM3.0 intends to complement not replace the existing approaches that are reference-based, partially-referenced, or reference-free.

Table S11. Functionality comparison among nine deconvolution methods (✔ - Yes; ✗ - No).

| Functionality | Detecting # of sources | Detecting novel source & markers | Estimating proportions | Estimating whole source profiles | Applicable data types |
| --- | --- | --- | --- | --- | --- |
| CAM3.0 (Chan, et al., 2008; Chen, et al., 2011; Wang, et al., 2016) | ✔ | ✔ | ✔ | ✔ | gene expression, proteomics, DNA methylation, medical imaging, metabolomics |
| Tsisal (Zhang, et al., 2021) | ✔ | ✔ | ✔ | ✔ | DNA methylation |
| TOAST (Li and Wu, 2019) | **✗** | **✗** | ✔ | ✔ | DNA methylation, gene expression |
| Houseman (Houseman, et al., 2016) | ✔ | **✗** | ✔ | ✔ | DNA methylation |
| TCA (Rahmani, et al., 2019) | **✗** | **✗** | **✗**  Proportion-based | ✔ | DNA methylation, gene expression |
| CIBERSORT (Newman, et al., 2015) | **✗** | **✗** | ✔ | ✔  Reference-based | gene expression |
| PREDE (Qin, et al., 2020) | ✔ | **✗** | ✔ | ✔  Partial reference-based | gene expression, concerned by (Avila Cobos, et al., 2020) |
| Semi-CAM (Dong, et al., 2020) | ✔ | ✔ | ✔ | ✔  Semi-supervised, w/ partial markers | gene expression |
| Linseed (Zaitsev, et al., 2019) | **✗** | ✔ | ✔ | ✔ | gene expression |

We then conducted experimental comparison to assess the performance of CAM3.0 and three unsupervised deconvolution methods (TOAST, Tsisal and Houseman) (Houseman, et al., 2016; Li and Wu, 2019; Zhang, et al., 2021) and one supervised deconvolution methods (TCA) (Rahmani, et al., 2019). The realistic simulation data were generated based on the two benchmark gene expression datasets (GSE73721 and GSE19380) and one benchmark DNA methylation dataset (GSE110554), where the mixing proportions were generated by the Dirichlet distributions with diverse parameter settings and varying numbers of samples, cell types and software runs. New experimental results show that CAM3.0 outperforms the peer methods in terms of higher column-wise correlation, higher row-wise correlation, and lower RMSE between the estimated proportion matrix (or cell type specific expression matrix) and the ground truth.

For the experimental comparison on gene expression data, we compared CAM3.0 with TOAST using the default setting given by the peer’s software package. The diverse mixing proportions were generated by the Dirichlet distributions with the following parameter settings: *K*=3: α1 = 4, α2 = 0.5, α3 = 4; *K*=4: α1 = 4, α2 = 0.5, α3 = 4, α4 = 0.5; *K*=5: α1 = 4, α2 = 0.5, α3 = 4, α4 = 0.5, α5 = 4. Experiments were repeated 50 times for each parameter setting. We acknowledge that while it is not feasible to test all possible parameter settings, these settings are justifiable because convenient completely random mixing is actually not biologically plausible. The comparative experimental results are summarized in Table S12.

Table S12. Experimental results in comparing CAM3.0 and TOAST in estimating the mixing proportion matrix on the realistically simulated gene expression data.


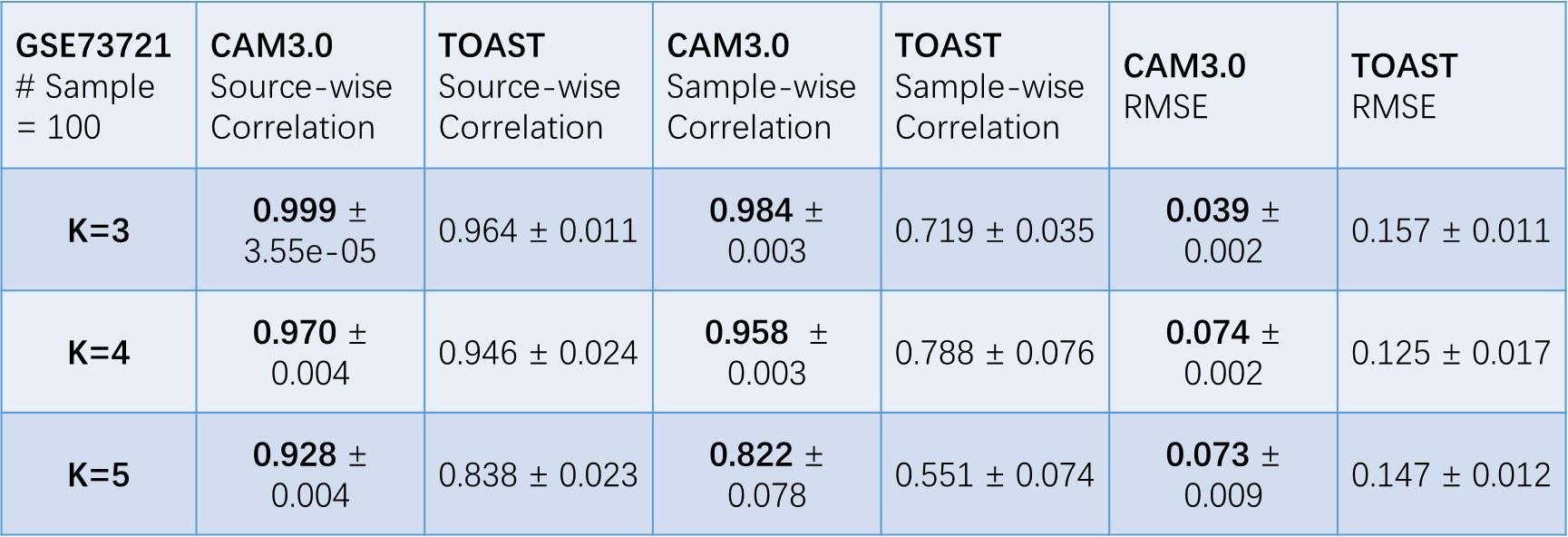


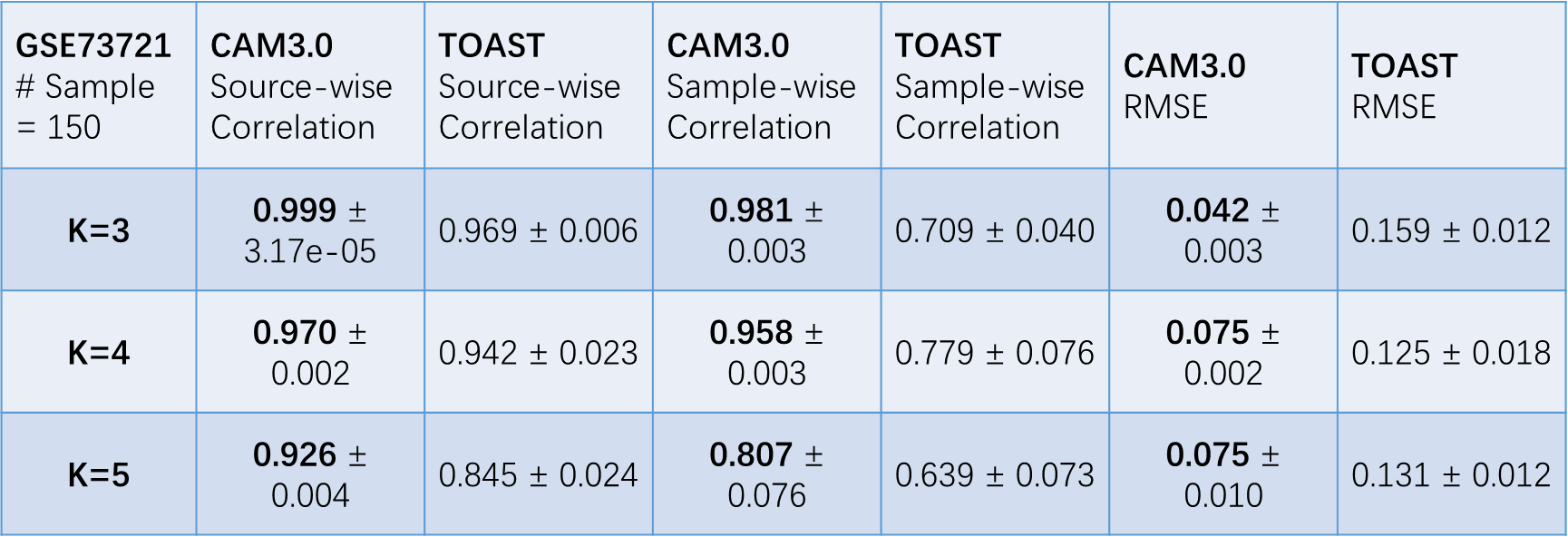


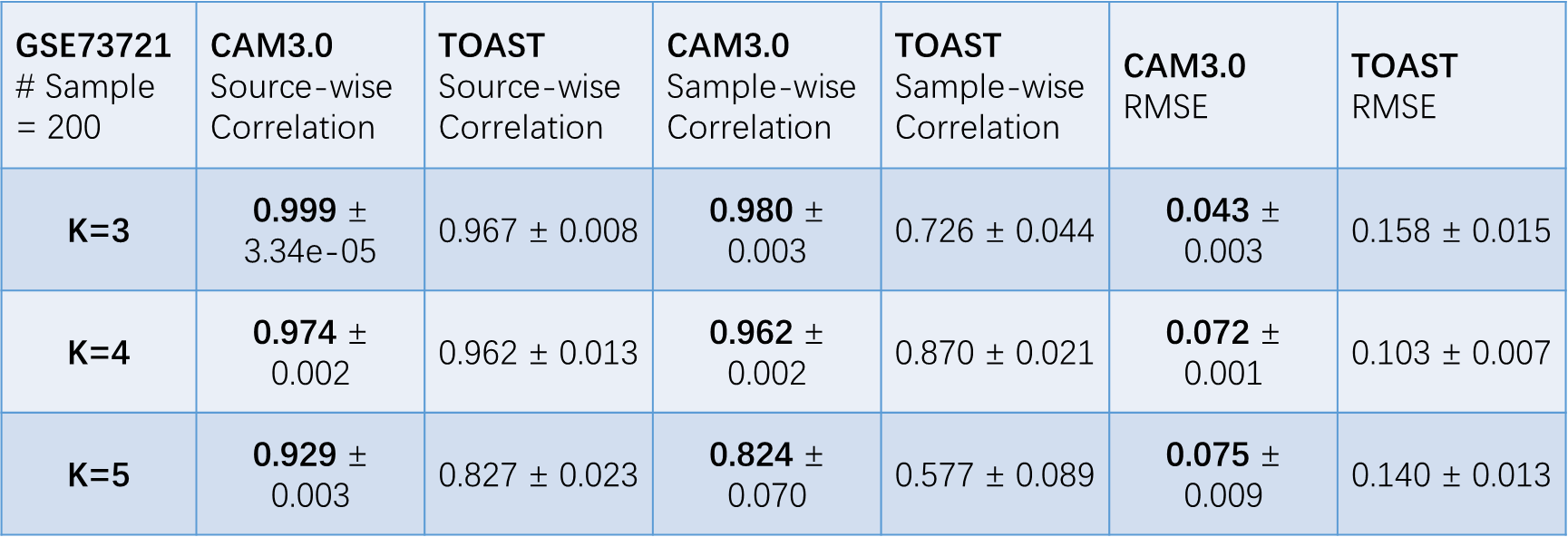


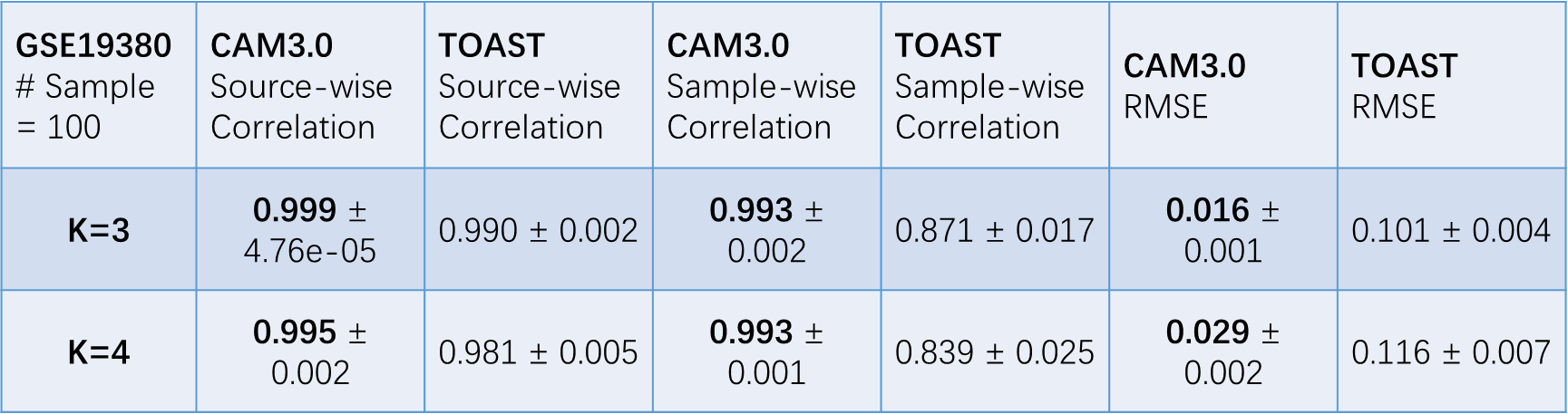


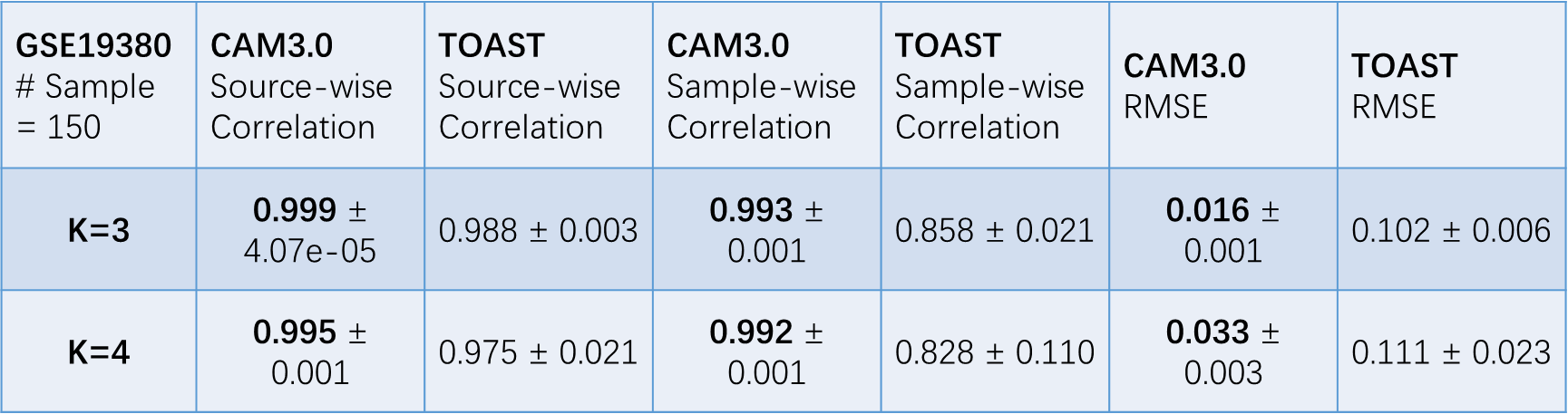


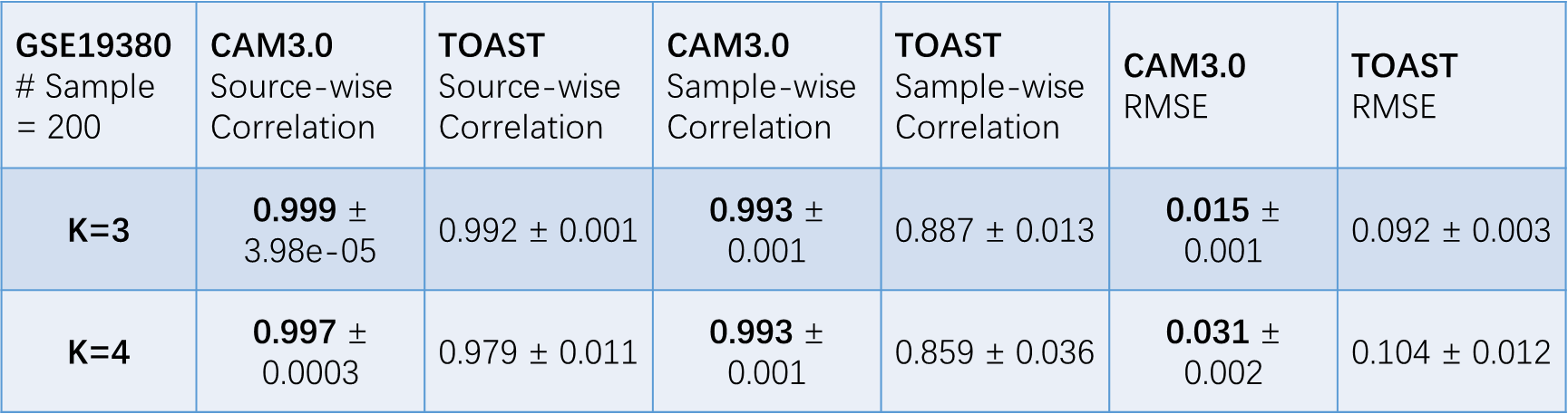


For the experimental comparison on DNA methylation data, we compared CAM3.0 with Tsisal and Houseman using the default settings given by the peers’ software packages. Mixing proportions were generated by the Dirichlet distributions with the following parameter settings: *K*=3: α1 = 2, α2 = 0.5, α3 = 2; *K*=4: α1 = 2, α2 = 0.5, α3 = 2, α4 = 0.5; *K*=5: α1 = 2, α2 = 0.5, α3 = 2, α4 = 0.5, α5 = 2. Experiments were repeated 50 times for each parameter setting. We acknowledge that while it is not feasible to test all possible parameter settings, these settings are reasonable because completely random mixing is actually not biologically plausible. The comparative experimental results are summarized in Table S13.

Table S13. Experimental results in comparing CAM3.0, Tsisal and Houseman in estimating the mixing proportion matrix on the realistically simulated DNA methylation data.


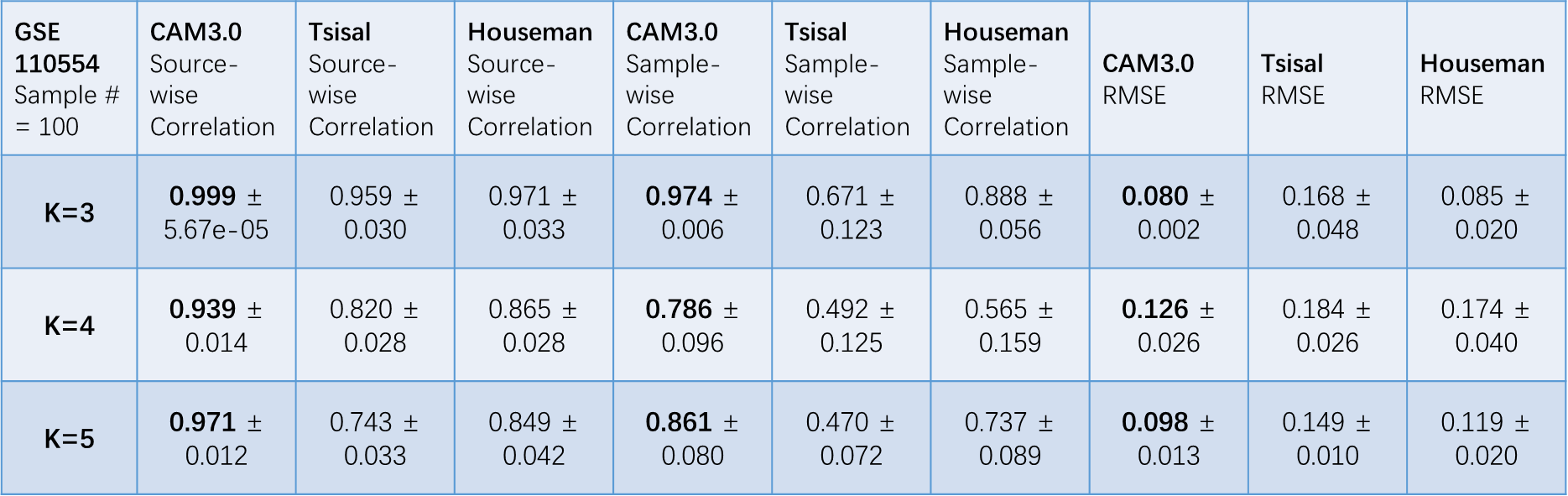


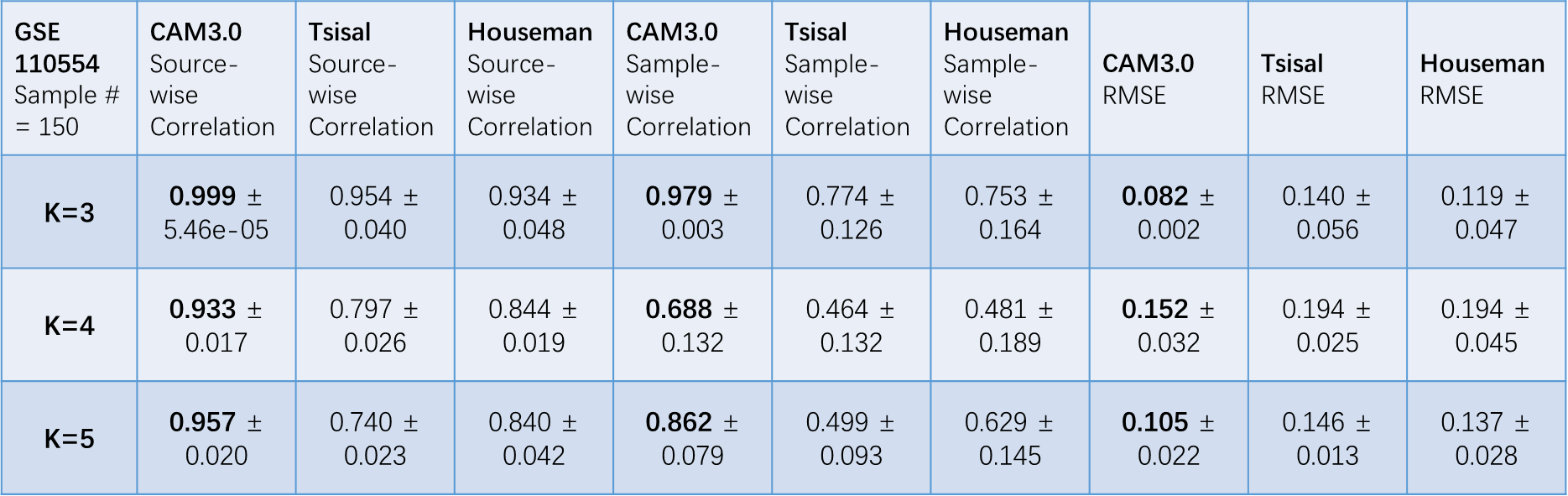


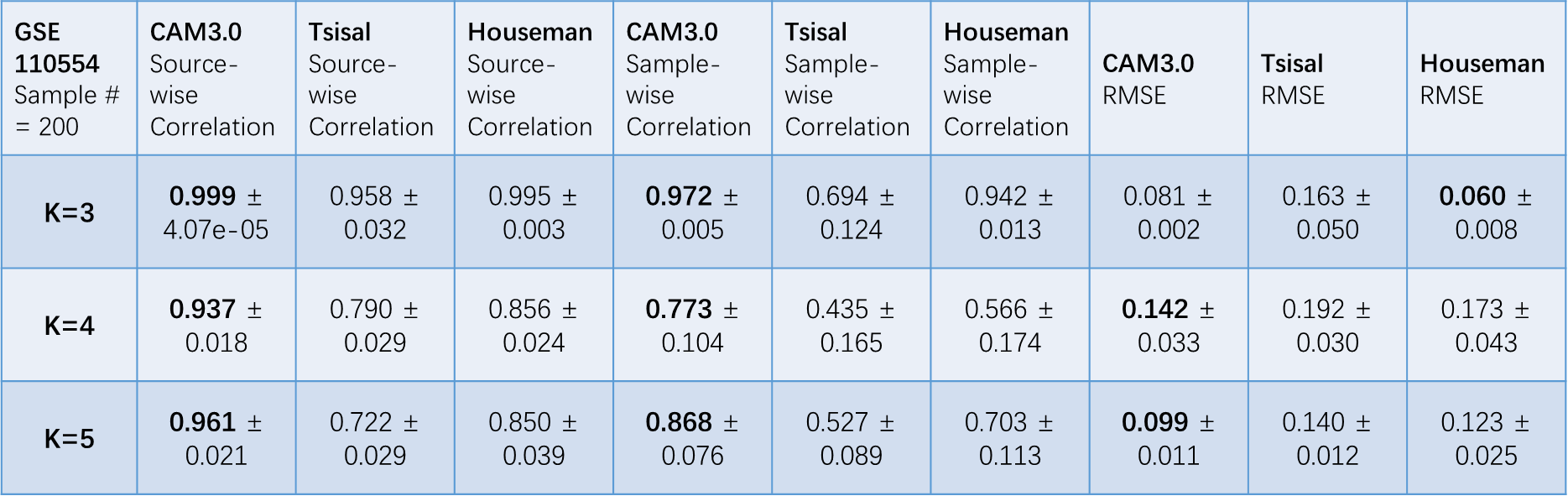


For the experimental comparison between CAM3.0 and TCA on gene expression data, while TCA has been considered not suitable for extensive comparative studies by some other researchers (Wang, et al., 2021), we have attempted to complete rational and fair comparisons. Baseline cell type specific expression profiles are sampled from the real gene expression in GSE73721. Specifically, 300 genes were picked randomly, with 20 simulations per experimental setting, default sample# = 100, MG# = 30, and *K*= 2, 3, 4, 5 with *K=4* as the default. Marker genes (MG) are chosen by the cosine score of cross-source expressions and ideal MG references. The variance of subtype expressions and variance of overall noise are set to be proportional to the means of subtype and mixed expressions, respectively. Performance accuracy is measured by RMSE, source-wise correlation (cell type), and gene-wise correlation, respectively. Results show that CAM3.0 performs comparably to TCA in estimating cell type-specific gene expression (Table S14a) when TCA is supervised by true mixing proportions. CAM3.0 outperforms TCA (Table S14b) when TCA is supervised by less accurate (noisy) mixing proportions. CAM3.0 also outperforms TCA (Table S14c) when TCA is supervised by incomplete mixing proportions with missing reference of one cell type (the accuracy is measured on only non-missing cell types). We have previously conducted and reported the similarly designed comparative experiments using the realistic simulations based on GSE19380 and obtained results consistent with those reported here (Chen, et al., 2022).

Table S14. Experimental results on comparing CAM3.0 and TCA in estimating cell type specific gene expression data matrix on the realistically simulated gene expression data (left-column: RMSE, middle-column: source-wise correlation, and right-column: gene-wise correlation).


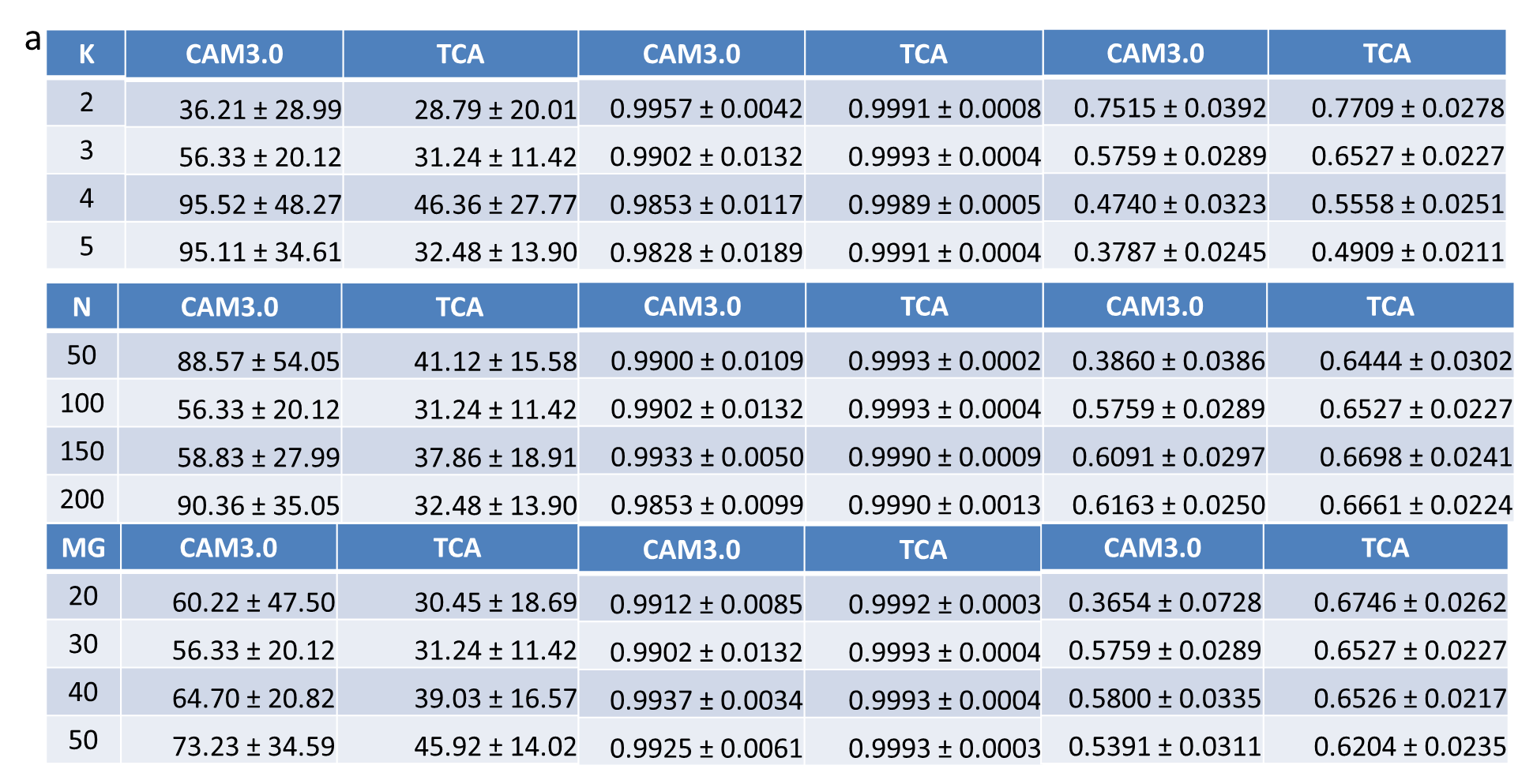


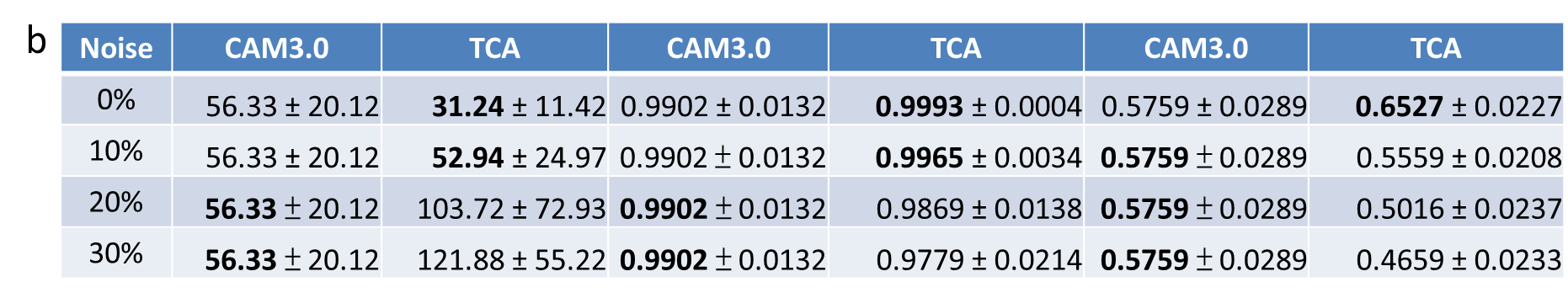


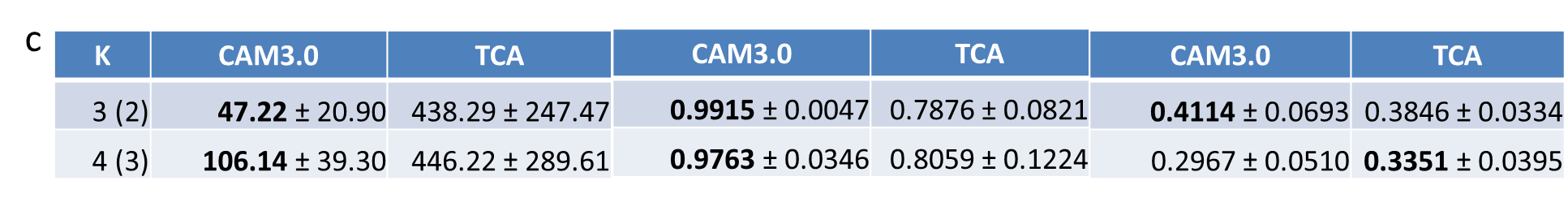


We also compared the accuracy of estimating the mixing proportion matrix, for the cases (GSE19380 microarray gene expression and GSE110554 DNA methylation) where the ‘biological’ ground truth is available, by CAM3.0, TOAST, Tsisal and Houseman. The comparative experimental results are summarized in Table S15 and Table S16.

Table S15. Experimental results on comparing CAM3.0 and TOAST in estimating cell type proportion matrix on the original microarray gene expression data (*K*=4).


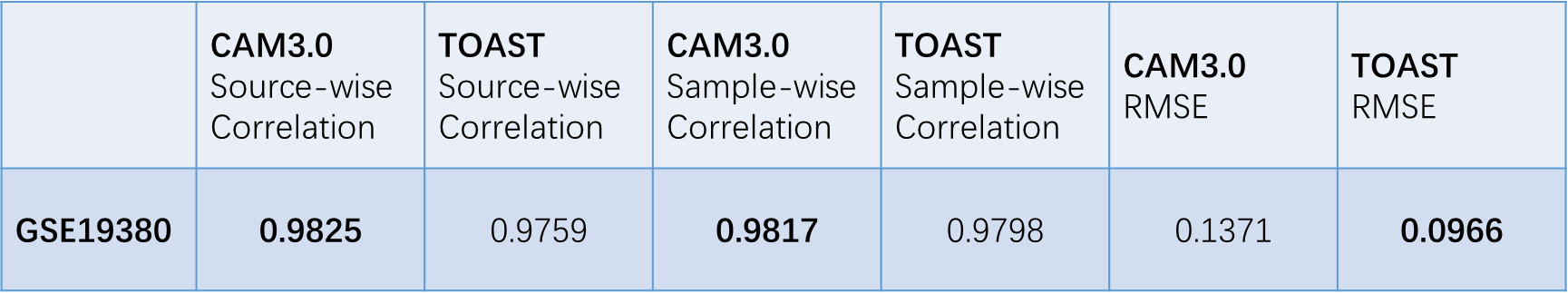


Table S16. Experimental results on comparing CAM3.0, Tsisal and Houseman in estimating cell type proportion matrix on the original benchmark DNA methylation data (*K*=6).


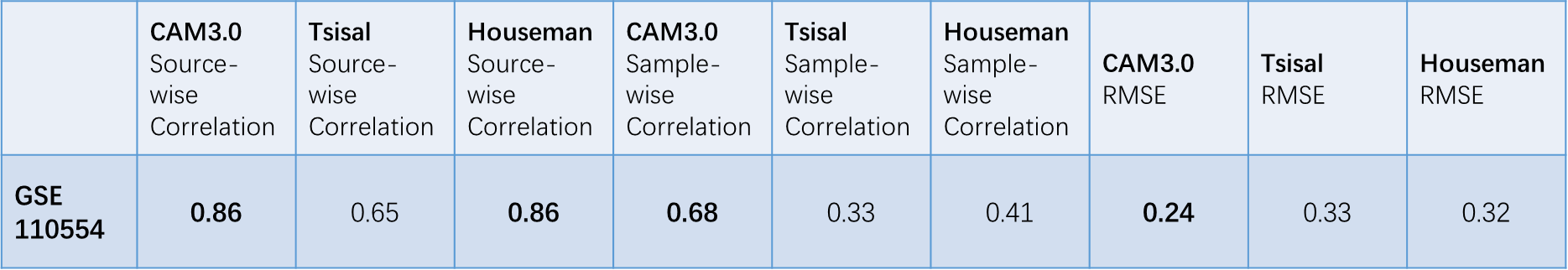


To re-assess the robustness to unseen cell types by reference-based deconvolutions (Newman, et al., 2015; Qin, et al., 2020), in addition to the previously reported comparisons using general references (noisy or study-non-specific) (Avila Cobos, et al., 2020; Chen, et al., 2022), we conducted new experimental evaluation. we employed snRNAseq and bulk-tissue RNAseq data collected from 35 matched postmortem brain samples to investigate the impact of absent cell types on the deconvolution accuracy. Specifically, we systematically excluded individual cell types from the reference set, generating partial references with one missing cell type. Subsequently, the deconvolution was carried out using the benchmark bMIND software based on these partial references (Wang, et al., 2021). We calculated Spearman correlation coefficients between the deconvoluted expression profiles and the referencing snRNAseq data obtained from the corresponding matched samples. Our findings revealed a notable reduction in correlation coefficients when major or moderate cell types (e.g. excitatory neurons) were omitted from the reference set (**Figure S13**). These results are consistent with the independent evaluation by Francisco Avila Cobos et. al stating that “Moreover, failure to include cell types in the reference that are present in a mixture leads to substantially worse results, regardless of the previous choices.” (Avila Cobos, et al., 2020).


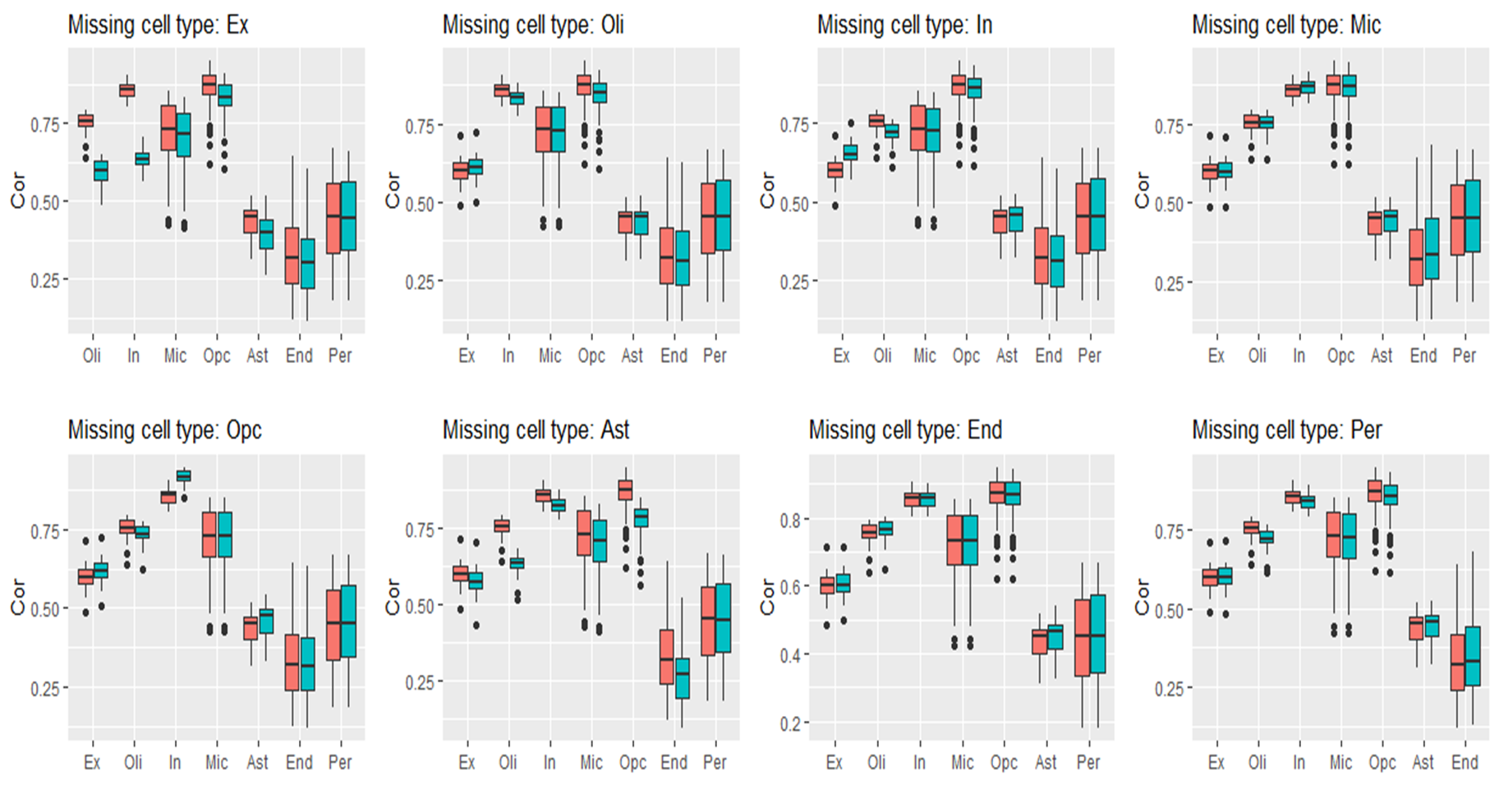


Figure S13. The impact of missing cell types on the performance of supervised deconvolution (bMIND) was assessed. Supervised deconvolution was conducted using both complete (in red) and incomplete (in blue) reference datasets on RNAseq data obtained from adult postmortem brains. The incomplete reference dataset was generated by excluding the expression data of one specific cell type. The resulting deconvoluted expressions were then correlated with the ground truth to evaluate the performance of the deconvolution method. Notably, the cell types included in the analysis were as follows: Excitatory Neurons (Ex), Inhibitory Neurons (In), Oligodendrocytes (Oli), Microglia (Mic), Oligodendrocyte Precursor Cells (Opc), Astrocytes (Ast), and Pericytes (Per).

Furthermore, we compared the cell composition in snRNAseq data and ground truth, as well as the pseudo bulk constructed from snRNAseq data and real bulk data. We used a data series from ROSMAP project, including snRNAseq, bulk RNAseq, and cell counting data from immunohistochemistry (IHC) of the same eight brain samples. Comparing to IHC data, snRNAseq captured more neurons and oligodendrocytes but less astrocyte, endothelial cells and microglia (**Figure S14A**). It suggests that snRNAseq technology may have cell-type-specific bias of cell capture. We next constructed pseudo bulk data with IHC proportions and snRNAseq proportions. Lower correlation with real bulk data was observed in pseudo-bulk constructed with snRNAseq proportions (**Figure S14B**). Even for the pseudo bulk constructed from ground truth IHC data, the median correlation to real bulk data is 0.48. It demonstrated that over half of information was missed in snRNAseq profile compared to bulk RNAseq.


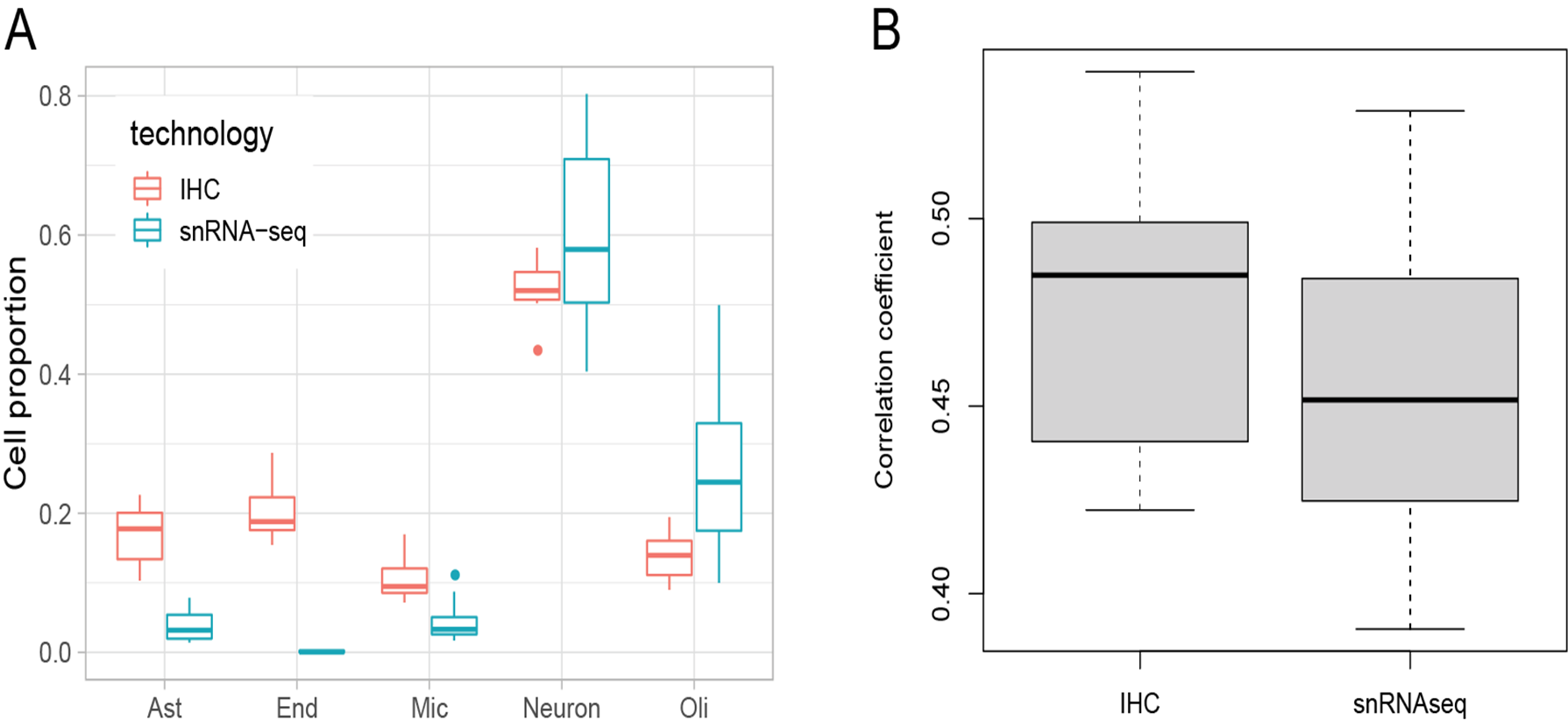


Figure S14. The limitations of snRNAseq data. (A) Comparison of cell proportions counted by immunohistochemistry (IHC) and proportions of cell types in snRNAseq data. (B) Spearman correlations between real bulk data and pseudo bulk data constructed from IHC proportions and snRNAseq proportions.

Another concern on using sn/scRNAseq references is that majority of genes in sn/scRNAseq data have high expression variability. Our assessment indicates an inverse correlation between expression variability and the quantity of sequenced cells within technical variations. When the cell count is below 1,500, over 50% of genes manifest a coefficient of variation exceeding 0.1, a threshold akin to that observed in conventional bulk-tissue RNAseq analyses. A similar observation was reported in (Wang, et al., 2021) stating that “Ideally, this would be evaluated by comparing scRNA-seq and CTS estimates obtained from the same samples, but in a comparison of bulk RNA-seq and reconstructed bulk expression obtained from snRNA-seq data, the per gene correlation was observed to be quite low.”

To assess the sensitivity-to-noise by CAM3.0 and the two most relevant peer methods (Tsisal and Houseman), we conducted experimental comparative evaluations on the performance of CAM3.0, Tsisal, and Housemen, subject to various levels of noise in the observed/input data. The realistic simulation data were generated based on the benchmark gene expression dataset (GSE73721; 15,401 features; *K*=4 cell types), where the mixing proportions were generated by the Dirichlet distributions with following parameter settings: *K*=4; α1 = 4; α2 = 1; α3 = 4; α4 = 1; 200 samples. The simulated observational additive noise follows a normal distribution with zero mean and standard deviation of 0.1, 0.2, 0.3, and 0.4, respectively; and then scaled up (proportionally normalized) by the expression means or averages. For each noise level (10%, 20%, 30%, 40%), 10 simulation data sets were randomly generated for the repeated experimental runs.

Evaluation criteria, on comparing the estimated proportion matrix and ground truth, include source-wise correlation, sample-wise correlation, and RMSE. The experimental parameter settings are: CAM3.0 (thres.low = 0.3, thres.high = 0.95, radius.thres = 0.995, cluster.num = 30, MG.num.thres = 5); Tsisal (Feature selection: nmarker = 5,000); and Houseman (Feature selection: selecting the most variant 10,000 features, verbose = FALSE). The comparative experimental results are summarized in Table S17. The experimental results show that CAM3.0 outperforms the peer methods in terms of higher column/source-wise correlation, higher row/sample-wise correlation, and lower RMSE between the estimated proportion matrix and the ground truth.

Table S17. Experimental results on sensitivity-to-noise comparing CAM3.0, Tsisal and Houseman in estimating the mixing proportion matrix.


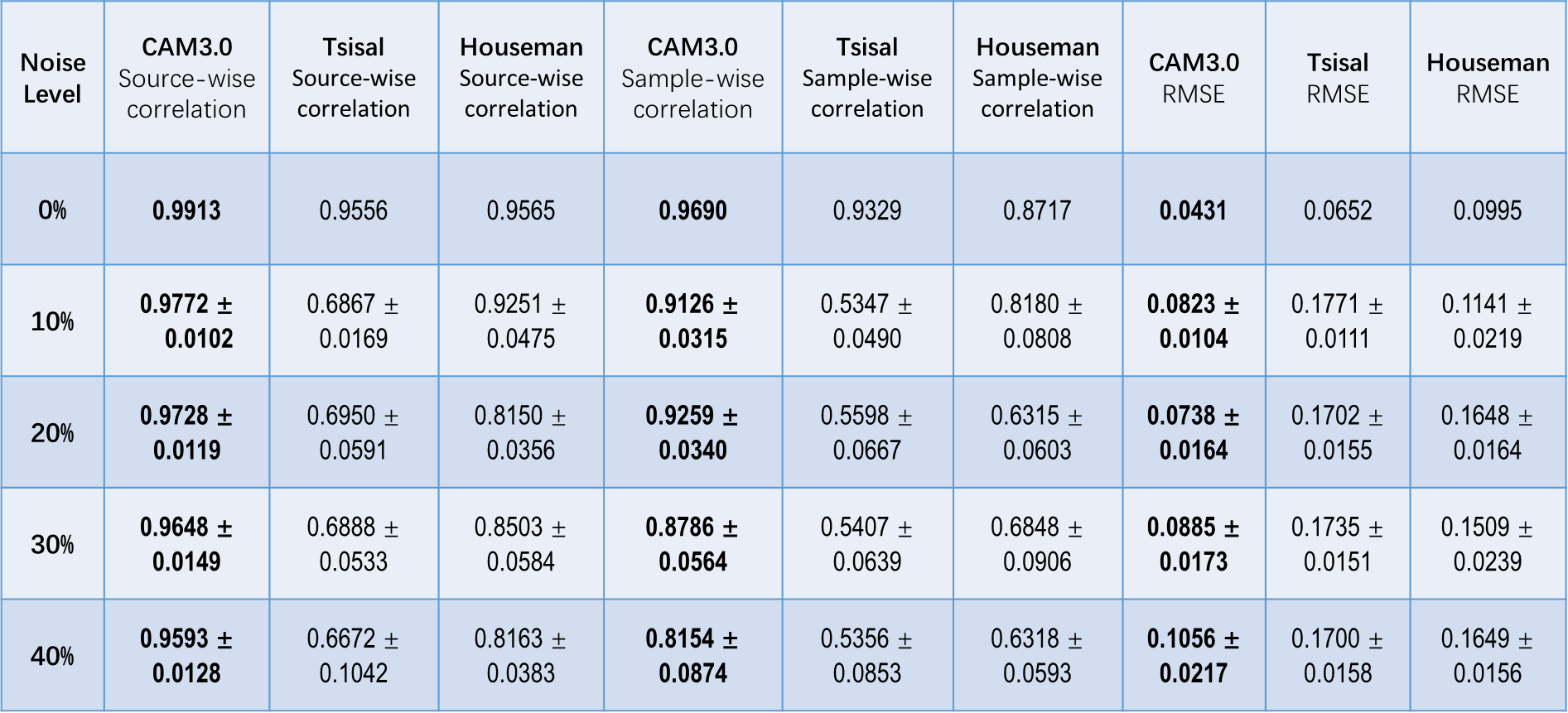


## Discussion

We report several methodological improvements to the CAM framework, evaluated and applied to performing fully unsupervised deep deconvolution. Principally, the quality of the latent variable model identified by CAM is determined jointly, based on a multi-term MDL criterion, by MGs (associated with the proportion matrix ***A***, with uniqueness and simplex rank) and by SGs (associated with source matrix ***S***, biologically interpretability and reconstruction error). In relation to the few unsupervised deconvolution methods, NMF seeks to produce a non-negative and sparse, yet non-unique and distribution-dependent solution, with limited biological interpretability; Pareto applies a similar principle as CAM while opted to exploit the sample-space (Chan, et al., 2008; Hart, et al., 2015; Wang, et al., 2010), requiring biologically-interpretable pure source samples with a much restricted applicability (architype samples are defined by the well-ground points in mixing matrix rather than source matrix). CAM3.0 outcomes readily provide complementary references for the supervised or semi-supervised deconvolution methods (Dong, et al., 2020; Qin, et al., 2020; Rahmani, et al., 2019). In particular, there is a strong collaborative relationship between CAM3.0 and semi-CAM (Dong, et al., 2020). While CAM3.0 is illustrated mainly on cell types, it is readily applicable to deconvolute bulk data into tissue types or biological tasks (Herrington, et al., 2018; Parker, et al., 2020). Note that CAM method has also been applied successfully to deconvolve functional medical imaging data (Chen, et al., 2011; Chen, et al., 2011; Chen, et al., 2014).

We would like to acknowledge and thank the pioneering work on bulk data deconvolution by Saidi et al (Saidi, et al., 2004) and Liebermeister (Liebermeister, 2002), who explored the use of independent component analysis and other tools for blind source separation. We should also comment on the noise effects in relation to the Linseed work by Zaitsev et al. (Zaitsev, et al., 2019). As aforementioned, RFC functional module is newly developed in CAM3.0 to identify quality cell type markers by scatter down-sampling while ensuring i) the tightness of gene clusters, ii) sufficient number of cluster members, iii) auto-removal of outliers, and iv) auto-determination of clusters numbers. The Linseed tool has previously been evaluated by Sutton et. al and Jaakkola et. al (Jaakkola and Elo, 2021; Sutton, et al., 2022). The evaluation results indicate that the model selection by SVD-score was inaccurate and several memory errors occurred with Linseed. Moreover, the original publication of Linseed focuses on estimating proportions and only a subset of cell type specific features that distinguishes among the cell types from each other, indicating that the method is not intended for full expression deconvolution (Jaakkola and Elo, 2021). A closer look at Linseed tool suggests that the method to identify robust scatter simplex may be suboptimum or even problematic. For example, Linseend tool also uses Sisal, a noise-dependent procedure, to identify simplex corners that is controlled by a hyperparameter tau: balancing between the feature inclusion and minimum volume. The choice of tau can produce very different simplex structures. It is unclear how the reconstruction accuracy can accurately determine the vertices without being influenced by the interior feature distribution independent of cell type markers, because the feature distribution varies with often asymmetry in marker distributions across cell types and some noise-like features are true markers, see below (left-2: examples used in Linseed, right: benchmark gene expression data GSE28490.


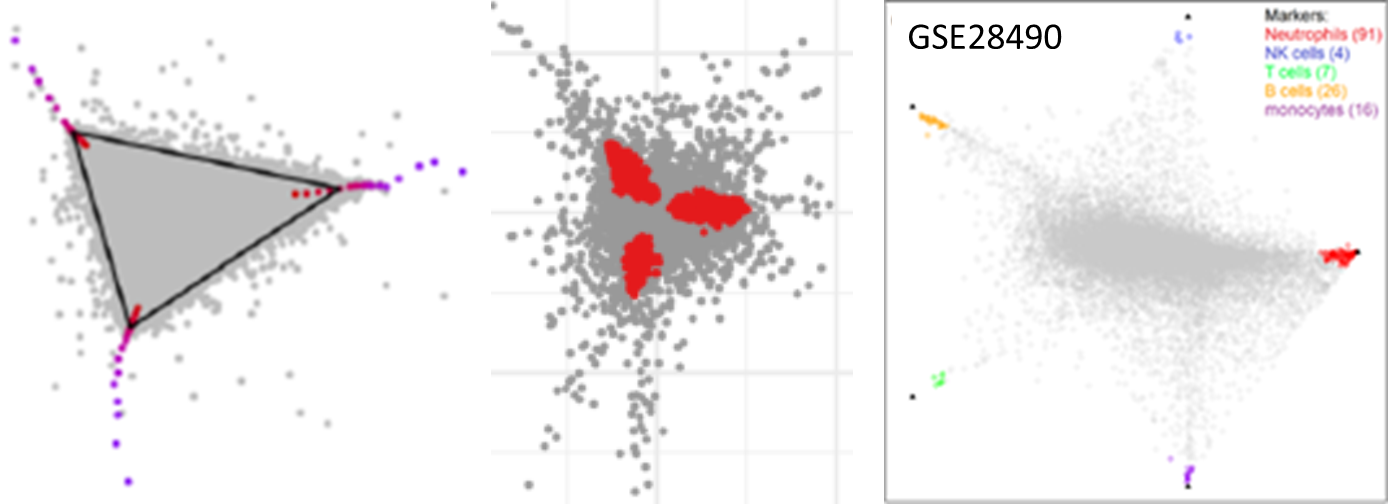


Concerning the appropriate application of the CAM method, the CAM version 3.0 is still imperfect and there remain opportunities for further improvements. First, while we can control the tightness of clusters to ensure the quality of MGs in the radius-fixed clustering, the computation time is expectedly much higher than K-means clustering. Because radius-fixed clustering forms clusters sequentially while K-means clustering determines clusters simultaneously, there is an opportunity to integrate these two approaches and leverage their advantages. For example, we can use K-means clustering to initialize the radius-fixed clustering, though the uncertainty of K-means clustering remains as a concern. Second, as an alternative to pre-clustering, we may adopt robust linear programming (Ben-Tal and Nemirovski, 2000) to find the convex hull with some randomness. Third, the identification of the optimal simplex may be formulated as a Lasso-type vertex selection problem (Yuan and Lin, 2006), where the feature points with significant coefficients will be selected as true or highly probable vertices while determining the appropriate penalty and hyperparameters remain an unresolved problem. Fourth, except for the clustering step, most other steps in the CAM3.0 pipeline are suitable for parallel computing. Fifth, as a fully unsupervised machine learning method, CAM3.0 is expectedly not immune to hidden confounding factors, e.g. batch effect, collinearity, and between-sample normalization and antilogarithm should be properly performed.

To determine and preserve the intrinsic scatter dimension (Chari, et al., 2021), a critical dimension reduction step for reducing the subsequent computational complexity, we adopt deep-learning based strategies (Hu, et al., 2017; Yang, et al., 2019), where the eigenvalues of data covariance matrix are used to estimate possible number of sources via a fully-connected deep neural network. In the CAM3.0 pipeline, we use the top 30 normalized singular values of the data matrix as the inputs and extensive simulation datasets to train the deep neural networks (**Fig. S4**). While this step aims to estimate the scatter dimension, we use twice the estimated dimensions as the upper limit for both PCA based dimension reduction and MDL based model selection to avoid the dangers of unrecoverable information loss due to dimensionality over-reduction. For readers interested in the impactful dimension reduction (Dong, et al., 2020), we highly recommend the topical discussions on the dangers of unsupervised dimensionality reduction to a very low number of dimensions, such as by the Pachter lab in their recent preprint (Chari, et al., 2021).

To achieve convexity and shape preserved simplex visualization, based on the method introduced in debCAM, we shall further propose another projection strategy that can retain the distances among vertices. Specifically, first the order of vertices on the circle is estimated according to the distance among the vertices, and then the arc length is determined by the distance between the directly neighboring vertices (Seth and Eugster, 2016). The steps of the newly proposed projection are as follows:

1. For the *K* vertices of the (*K*-1)-simplex, find the three points (for example, $b$, $d$, and $f$) which form the triangle with the maximum area among all possible $C_{3}^{K}$ combinations exhaustively.
2. After finding out the three points, project them onto a circle temporarily ($b^{'}$, $d^{'}$, and $f^{'}$), note that the arc length is not important in this step.
3. For the remaining *K*-3 points, sort them by the sum of distance to the three points ($b$, $d$, and $f$) detected in the first step decreasingly in a list.
4. According to the order after sorting in the third step, find the first point (for example, $a$), and compare the distance between this point ($a$) to the points not in the list ($b$, $d$, and $f$). Find out the two points which are the closest to the point ($d$ and $f$), and then the point is projected onto the arc between the projection of the two points ($a^{'}$ is between $d^{'}$ and $f^{'}$). Remove the point from the list.
5. Repeat the fourth step until there is no points left in the list.

Assign the arc length by the distance of the two end points of each arc before projection (in the original space).

## R Scripts

The prototype R scripts are available at <https://github.com/ChiungTingWu/CAM3/>, (https://github.com/Bioconductor/Contributions/issues/3205)

More suggestions on parameter setting can be found in the package vignette.

## References

Avila Cobos, F.*, et al.* Benchmarking of cell type deconvolution pipelines for transcriptomics data. *Nat Commun* 2020;11(1):5650.

Avila Cobos, F.*, et al.* Computational deconvolution of transcriptomics data from mixed cell populations. *Bioinformatics* 2018;34(11):1969-1979.

Azevedo, F.A.*, et al.* Automatic isotropic fractionation for large-scale quantitative cell analysis of nervous tissue. *J Neurosci Methods* 2013;212(1):72-78.

Azevedo, F.A.*, et al.* Equal numbers of neuronal and nonneuronal cells make the human brain an isometrically scaled-up primate brain. *J Comp Neurol* 2009;513(5):532-541.

Bakay, M.*, et al.* Nuclear envelope dystrophies show a transcriptional fingerprint suggesting disruption of Rb-MyoD pathways in muscle regeneration. *Brain* 2006;129(Pt 4):996-1013.

Barber, C.B., Dobkin, D.P. and Huhdanpaa, H. The quickhull algorithm for convex hulls. *ACM Transactions on Mathematical Software (TOMS)* 1996;22(4):469-483.

Ben-Tal, A. and Nemirovski, A. Robust solutions of linear programming problems contaminated with uncertain data. *Mathematical programming* 2000;88(3):411-424.

Chan, T.-H.*, et al.* A convex analysis framework for blind separation of non-negative sources. *IEEE Trans Signal Processing* 2008;56(10):5120-5134.

Chari, T., Banerjee, J. and Pachter, L. The specious art of single-cell genomics. *bioRxiv* 2021:doi.org/10.1101/2021.1108.1125.457696.

Chen, L.*, et al.* CAM-CM: a signal deconvolution tool for in vivo dynamic contrast-enhanced imaging of complex tissues. *Bioinformatics* 2011;27(18):2607-2609.

Chen, L.*, et al.* Tissue-specific compartmental analysis for dynamic contrast-enhanced MR imaging of complex tumors. *IEEE Trans Med Imaging* 2011;30(12):2044-2058.

Chen, L.*, et al.* Unsupervised Deconvolution of Dynamic Imaging Reveals Intratumor Vascular Heterogeneity and Repopulation Dynamics. *PLoS One* 2014;9(11):e112143.

Chen, L.*, et al.* Data-driven robust detection of tissue/cell-specific markers. *bioRxiv* 2019:517961.

Chen, L.*, et al.* swCAM: estimation of subtype-specific expressions in individual samples with unsupervised sample-wise deconvolution. *Bioinformatics* 2022;38(5):1403-1410.

Chen, L.*, et al.* debCAM: a bioconductor R package for fully unsupervised deconvolution of complex tissues. *Bioinformatics* 2020;36(12):3927-3929.

Colantuoni, C.*, et al.* Temporal dynamics and genetic control of transcription in the human prefrontal cortex. *Nature* 2011;478(7370):519-523.

Colantuoni, C.*, et al.* Temporal dynamics and genetic control of transcription in the human prefrontal cortex. *Nature* 2011;478(7370):519-523.

Dadgar, S.*, et al.* Asynchronous remodeling is a driver of failed regeneration in Duchenne muscular dystrophy. *J Cell Biol* 2014;207(1):139-158.

Dong, L.*, et al.* Semi-CAM: A semi-supervised deconvolution method for bulk transcriptomic data with partial marker gene information. *Sci Rep* 2020;10(1):5434.

Fan, M.*, et al.* Radiogenomic signatures reveal multiscale intratumour heterogeneity associated with biological functions and survival in breast cancer. *Nat Commun* 2020;11(1):4861.

Gaujoux, R. and Seoighe, C. Semi-supervised Nonnegative Matrix Factorization for gene expression deconvolution: a case study. *Infect Genet Evol* 2012;12(5):913-921.

Gaujoux, R. and Seoighe, C. CellMix: a comprehensive toolbox for gene expression deconvolution. *Bioinformatics* 2013;29(17):2211-2212.

Hart, Y.*, et al.* Inferring biological tasks using Pareto analysis of high-dimensional data. *Nat Methods* 2015;12(3):233-235.

Herrington, D.M.*, et al.* Proteomic Architecture of Human Coronary and Aortic Atherosclerosis. *Circulation* 2018;137(25):2741-2756.

Houseman, E.A.*, et al.* Reference-free deconvolution of DNA methylation data and mediation by cell composition effects. *BMC Bioinformatics* 2016;17:259.

Hu, W.*, et al.* A deep learning method to estimate independent source number. In, *2017 4th International Conference on Systems and Informatics (ICSAI)*. IEEE; 2017. p. 1055-1059.

Jaakkola, M.K. and Elo, L.L. Computational deconvolution to estimate cell type-specific gene expression from bulk data. *NAR Genom Bioinform* 2021;3(1):lqaa110.

Kang, H.J.*, et al.* Spatio-temporal transcriptome of the human brain. *Nature* 2011;478(7370):483-489.

Kelley, R. and Ideker, T. Systematic interpretation of genetic interactions using protein networks. *Nature biotechnology* 2005;23(5):561-566.

Krug, K.*, et al.* Proteogenomic Landscape of Breast Cancer Tumorigenesis and Targeted Therapy. *Cell* 2020;183(5):1436-1456 e1431.

Kuhn, A.*, et al.* Population-specific expression analysis (PSEA) reveals molecular changes in diseased brain. *Nat Methods* 2011;8(11):945-947.

Lake, B.B.*, et al.* Neuronal subtypes and diversity revealed by single-nucleus RNA sequencing of the human brain. *Science* 2016;352(6293):1586-1590.

Li, Z. and Wu, H. TOAST: improving reference-free cell composition estimation by cross-cell type differential analysis. *Genome Biol* 2019;20(1):190.

Liebermeister, W. Linear modes of gene expression determined by independent component analysis. *Bioinformatics* 2002;18(1):51-60.

Lin, C.H.*, et al.* Detection of Sources in Non-Negative Blind Source Separation by Minimum Description Length Criterion. *IEEE Trans Neural Netw Learn Syst* 2018;29(9):4022-4037.

MacQueen, J. Some methods for classification and analysis of multivariate observations. In, *Proceedings of the fifth Berkeley symposium on mathematical statistics and probability*. Oakland, CA, USA; 1967. p. 281-297.

Mancarci, B.O.*, et al.* Cross-Laboratory Analysis of Brain Cell Type Transcriptomes with Applications to Interpretation of Bulk Tissue Data. *eNeuro* 2017;4(6).

Moffitt, R.A.*, et al.* Virtual microdissection identifies distinct tumor- and stroma-specific subtypes of pancreatic ductal adenocarcinoma. *Nat Genet* 2015;47(10):1168-1178.

Newman, A.M.*, et al.* Robust enumeration of cell subsets from tissue expression profiles. *Nat Methods* 2015;12(5):453-457.

Oba, S.*, et al.* A Bayesian missing value estimation method for gene expression profile data. *Bioinformatics* 2003;19(16):2088-2096.

Pardalos, P.M., Li, Y. and Hager, W. Linear programming approaches to the convex hull problem in Rm. *Computers & Mathematics with Applications* 1995;29(7):23-29.

Parker, S.J.*, et al.* Identification of Putative Early Atherosclerosis Biomarkers by Unsupervised Deconvolution of Heterogeneous Vascular Proteomes. *J Proteome Res* 2020;19(7):2794-2806.

Pudil, P., Novovičová, J. and Kittler, J. Floating search methods in feature selection. *Pattern recognition letters* 1994;15(11):1119-1125.

Qin, Y.*, et al.* Deconvolution of heterogeneous tumor samples using partial reference signals. *PLoS Comput Biol* 2020;16(11):e1008452.

Rahmani, E.*, et al.* Cell-type-specific resolution epigenetics without the need for cell sorting or single-cell biology. *Nat Commun* 2019;10(1):3417.

Saidi, S.A.*, et al.* Independent component analysis of microarray data in the study of endometrial cancer. *Oncogene* 2004;23(39):6677-6683.

Schwartz, R. and Shackney, S.E. Applying unmixing to gene expression data for tumor phylogeny inference. *BMC Bioinformatics* 2010;11:42.

Seth, S. and Eugster, M.J.A. Probabilistic archetypal analysis. *Machine Learning* 2016(102):85-113.

Shen-Orr, S.S.*, et al.* Cell type–specific gene expression differences in complex tissues. *Nature methods* 2010;7(4):287-289.

Stekhoven, D.J. and Bühlmann, P. MissForest—non-parametric missing value imputation for mixed-type data. *Bioinformatics* 2012;28(1):112-118.

Stiles, J. and Jernigan, T.L. The basics of brain development. *Neuropsychol Rev* 2010;20(4):327-348.

Sutton, G.J.*, et al.* Comprehensive evaluation of deconvolution methods for human brain gene expression. *Nat Commun* 2022;13(1):1358.

Wang, F.Y.*, et al.* Nonnegative least-correlated component analysis for separation of dependent sources by volume maximization. *IEEE Trans Pattern Anal Mach Intell* 2010;32(5):875-888.

Wang, J., Roeder, K. and Devlin, B. Bayesian estimation of cell type-specific gene expression with prior derived from single-cell data. *Genome Res* 2021.

Wang, N.*, et al.* Mathematical modelling of transcriptional heterogeneity identifies novel markers and subpopulations in complex tissues. *Scientific Reports* 2016;6:18909.

Wax, M. and Kailath, T. Detection of signals by information theoretic criteria. *IEEE Transactions on acoustics, speech, and signal processing* 1985;33(2):387-392.

Xu, X., Nehorai, A. and Dougherty, J.D. Cell type-specific analysis of human brain transcriptome data to predict alterations in cellular composition. *Systems Biomedicine* 2013;1(3):0--1.

Yang, Y.*, et al.* Model-aided deep neural network for source number detection. *IEEE Signal Processing Letters* 2019;27:91-95.

Yuan, M. and Lin, Y. Model selection and estimation in regression with grouped variables. *Journal of the Royal Statistical Society: Series B (Statistical Methodology)* 2006;68(1):49-67.

Zaitsev, K.*, et al.* Complete deconvolution of cellular mixtures based on linearity of transcriptional signatures. *Nat Commun* 2019;10(1):2209.

Zhang, W., Wu, H. and Li, Z. Complete deconvolution of DNA methylation signals from complex tissues: a geometric approach. *Bioinformatics* 2021;37(8):1052-1059.

Zhu, Y.*, et al.* Convex Analysis of Mixtures for Separating Non-negative Well-grounded Sources. *Sci Rep* 2016;6:38350.
